# Integrated RNA-Seq Analysis Uncovers the Potential Mechanism of the “Kidney Governing Bones” Theory of TCM

**DOI:** 10.1155/2022/7044775

**Published:** 2022-03-30

**Authors:** Shuo Yang, Tiancheng Wang, Jingcheng Zhang, Xiangyang Leng, Baojin Yao

**Affiliations:** ^1^Changchun University of Chinese Medicine, Changchun 130117, China; ^2^Jilin Ginseng Academy, Changchun University of Chinese Medicine, Changchun 130117, China

## Abstract

**Background:**

As in philosophy of traditional Chinese medicine (TCM), the theory of “kidney governing bones” has been demonstrated by a series of scientific studies. Furthermore, many groups including ours have explored the molecular mechanisms related to bone development, growth, and regeneration using modern biology technologies, such as RNA sequencing (RNA-Seq) and isobaric tags for relative and absolute quantification (ITRAQ), and have demonstrated that the underlying molecular mechanisms were highly consistent with the “kidney governing bones” theory.

**Objective:**

Kidney-yang deficiency (YD), as a pathological condition, has a passive effect on the skeleton growth; more specifically, it is a state of skeletal metabolic disorder. However, the exact molecular mechanisms related to the “kidney governing bones” theory under the control of multiple organs and systems are still unknown.

**Methods:**

In this study, we performed RNA-Seq analysis to investigate and compare the gene expression patterns of six types of tissue (bone, cartilage, kidney, testicle, thyroid gland, and adrenal gland) from YD rats and normal rats and analyzed the interaction effects controlled by multiple functional genes and signaling pathways between those tissues.

**Results:**

Our results showed that, in the state of YD, the functions of bone and cartilage were inhibited. Furthermore, multiple organs involving the reproductive, endocrine, and urinary systems were also investigated, and our results showed that YD could cause dysfunctions of these systems by downregulating multiple functional genes and signaling pathways that positively regulate the homeostasis of these tissues.

**Conclusion:**

We ensure that “kidney governing bones” was not a simple change in a single gene but the changes in complex biological networks caused by functional changes in multiple genes. This also coincides with the holistic view of TCM, which holds that the human body itself is an organic whole and the functional activities of each organ coordinate with each other.

## 1. Introduction

In traditional Chinese medicine (TCM), the conception of the relationship between kidney and bone based on “The Yellow Emperor's Internal Classic (Huang Di Nei Jing)” has been neatly summarized as “kidney governing bones.” Specifically, Kidney-Jing, the substance of the so-called kidney essence, has the potential to transform into marrow for the maintenance of normal bone growth, development, and regeneration, indicating Kidney-Jing is associated with bone metabolism. “Yin and yang in balance” as a TCM conception is the dynamic equilibrium of the physiological processes. As in TCM philosophy, the balance between yin and yang ultimately ensures homeostasis and a healthy cell or organism, including the skeleton. As a result of an imbalance of yin and yang, kidney deficiency as a pathological condition negatively affects the skeleton, resulting to bone metabolism disorders. To restore the kidney essence for the regulation of disorders of yin-yang in the kidney and to restore the balance of “yin and yang” within the body become a basic approach for the treatment of bone-related diseases. “Kidney-yang deficiency syndrome,” one of the common syndromes in TCM, was noted first in an early methodical and theoretical monograph existing in China called “Neijing,” and the features were warm dysfunction and metabolism-related disorder of body fluid, cold limbs, distaste to cold, etc. [1, 2]. Studies have proved that the main organ system altered in YD is the hypothalamus-pituitary-target gland (adrenal, thyroid, and gonad) axis to diverse degrees in various functional disorders [3, 4]. The hypothalamus-pituitary-target gland axis is a term describing the complicated, dynamic, and often regulatory interplays involving multiple glands: the hypothalamus, the pituitary gland, and the adrenal gland. It also includes the thyroid, testicle, and kidney. They regulate many physical processes in the body, including bone metabolism. According to the explanation of the kidney in TCM concepts, which is distinct from modern medicine, the kidney is a comprehensive organ that is not just reputed as an anatomical structure. In TCM, the kidney's duties include storing and controlling “essence” and regulating growth and development. This means that the kidneys are responsible for the skeleton's growth and repair [5]. Previously, our group [6–10] explored the molecular mechanisms related to bone development, growth, and regeneration using modern biology technologies, such as RNA sequencing (RNA-Seq), and demonstrated that the underlying molecular mechanisms were highly consistent with the “kidney governing bones” theory. However, the precise molecular mechanism underlying “kidney governing bones” is still not clearly understood. As we know, the majority of diseases are not caused by alters in a single gene but rather by the interruption of biological networks caused by the dysfunction of some genes or their products. In the present study, our team performed RNA-Seq to analyze the gene expression patterns of the bone, cartilage, kidney, testicle, thyroid, and adrenal gland in Sprague Dawley (SD) rats (kidney-yang-deficient rats and normal rats). After a comprehensive analysis of our sequencing results, we found that “kidney governing bones” resulted in a change in biological networks caused by the functional change of many genes or their products. It is well known that the kidneys, testes, and thyroid and adrenal gland are involved in the functions of the genitourinary and endocrine systems. This change of biological networks should be the result of the coordinated regulation of the endocrine urogenital system. This also coincides with the holistic view of traditional Chinese medicine (TCM) that the human body itself is an organic whole and the functional activities of each organ coordinate with each other and affect each other pathologically.

## 2. Materials and Methods

### 2.1. Animal Care and Experiments

Twenty male SD rats (6 weeks old, 180–220 g each, SPF grade) were obtained from the Liaoning Chang Sheng Biotechnology Co, Ltd. (Liaoning, China). The experimental animal certification number was SCXK (Liao) 2020-0001. The rats were kept in a temperature-controlled environment (temperature 22 ± 2°C, relative humidity 50 ± 10%) under a 12-h/12-h light/dark cycle. The Changchun University of Chinese Medicine's Institutional Animal Care and Use Committee approved all experimental protocols, and all experiments were executed in accordance with relevant guidelines and regulations. Prior to the experiment, the animals were given a week to acclimate to their new surroundings. Each rat was randomly assigned to one of the two groups of ten rats: a control group and a YD model group. Rats in the control group were given an intramuscular injection of 0.3 mL 0.9% saline into hind limbs, once a day, for 22 days continuously. For the YD model group, a 2.5 mg/100 g hydrocortisone suspension was injected intramuscularly into hind limbs, once a day, for 22 days continuously.

### 2.2. Sample Collection

The rats were sacrificed in batches. The thyroid gland, adrenal gland, testicle, kidney, bone, and cartilage were gathered in the morning following injection for 22 days. To summarize, one animal at a time was euthanized with CO_2_, and blood was drawn from the abdominal aorta. Following that, a cervical dislocation was performed to ensure death. Femurs were gathered by removing muscles, ligaments, and any other irrelevant tissues from both sides of each rat and stored at −80°C for RNA extraction. Meanwhile, cartilage, kidney, testicle, adrenal gland, and thyroid tissues from both sides of each rat were collected and stored at −80°C for RNA extraction. The upper serum was taken and stored at −20°C in a refrigerator after centrifugation (3000 rpm for 10 min, 4°C) for biochemical analysis via enzyme-linked immunosorbent assay (ELISA). The biochemical indicators used included ACTH, CORT, T3, T4, FSH, and T. Meanwhile, the thyroid gland, adrenal gland, testicle, kidney, bone, and cartilage of all the rats were collected for histopathological analyses. Briefly, the thyroid gland, adrenal gland, kidney, testicle, bone, and cartilage were fixed overnight with 10% formalin solution, while testicular tissues were fixed overnight with Bouin's fluid. Tissues were then fixed in paraffin wax and sectioned into 3 mm pieces using a microtome. After that, all the tissue sections were stained with hematoxylin and eosin (H&E) and photographed under an optical microscope to confirm the results.

### 2.3. RNA Isolation and Illumina Sequencing

Bones, cartilages, kidneys, testicles, thyroids and adrenal glands from each group were pooled together and ground into a fine powder, respectively. In other words, we pooled the thyroid of the control group and the YD group separately. Total RNA was extracted according to the manufacturer's instructions using the TRIzol reagent (Invitrogen, USA). RNA quality was determined by calculating RNA integrity number (RIN) using an Agilent 2100 bioanalyzer (Agilent Technologies, USA). Paired-end mRNA libraries were prepared using the TruSeq Stranded mRNA kit (Illumina, USA) according to the manufacturer's protocol. Transcriptome sequencing by RNA-Seq was carried out on Illumina HiSeq 2500 (Illumina, USA).

### 2.4. RNA-Seq Data Analysis

Clean readings were acquired following RNA-Seq by filtering out low-quality reads and adaptor sequences. HISAT was used to map the clean reads from each sample to the rat reference genome [11]. Gene expression levels were calculated by the FPKM algorithm [12]. BLAST was used to execute annotations against the nonredundant (NR) and Swiss-Prot protein databases. The R package DEGseq was used to identify differentially expressed genes [13]. Genes with a log_2_ fold change ≥1 or ≤−1 and with a *p* value ≤0.001 were considered to be differentially expressed genes (DEGs).

### 2.5. Function and Pathway Enrichment Analyses of Differentially Expressed Genes

Gene ontology (GO) and Kyoto Encyclopedia of Genes and Genomes (KEGG) enrichment analyses were carried out using the R package phyper. In the enrichment analysis, we used the hypergeometric test and the Bonferroni correction. After multiple testing corrections, the GO terms or pathways with a corrected *p* value (Q value) less than 0.05 were considered significantly enriched in our set of DEGs [14].

### 2.6. Validation by qRT-PCR

qRT-PCR was used to confirm the expression levels of DEGs identified from RNA-Seq analysis. In short, the total RNA used for RNA-Seq was used as a templet for cDNA synthesis via the iScript cDNA synthesis kit (Bio-Rad, USA) and subsequently amplified on the CFX Connect Real-Time PCR Detection System (Bio-Rad, USA) by using the SsoAdvanced Universal SYBR® Green Supermix (Bio-Rad, USA) in accordance with the manufacturer's instructions. The relative levels of mRNA expression were determined using the 2^−ΔΔCT^ algorithm, and the rat glyceraldehyde 3-phosphate dehydrogenase gene (*Gapdh*) was used as a reference gene for normalization [15].

## 3. Results

### 3.1. Weight Change, Biochemical Parameter Values, and Changes in Histology

**Figure 1:**
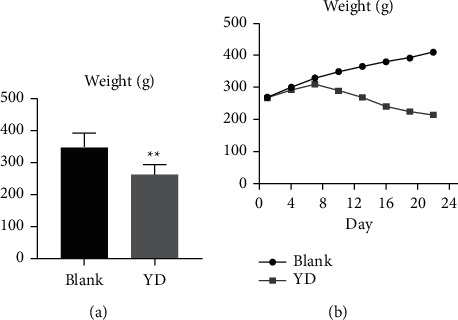
(a) Changes in the body weight index of rats (blank group and YD group) on day 22. (b) Changes in the body weight index of rats (blank group and YD group) every three days. The weight of rats in the blank group continued to increase, while the weight of rats in the YD group increased slowly over the first 7 days and then decreased from the 7th day, resulting in a negative weight increase.

**Figure 2:**
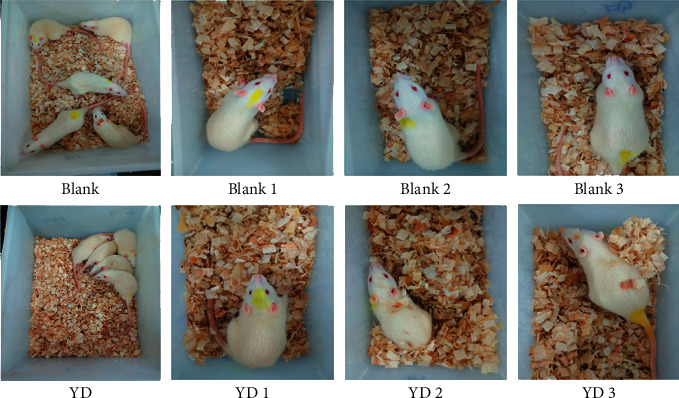
Morphological changes in YD rats. Compared with the blank group, YD rats developed a series of behavioral and physiological characteristics, including fatigue, reduced activity, the tendency to cluster, an aversion to cold, chills, a drop in appetite, and loss of hair.

**Figure 3:**
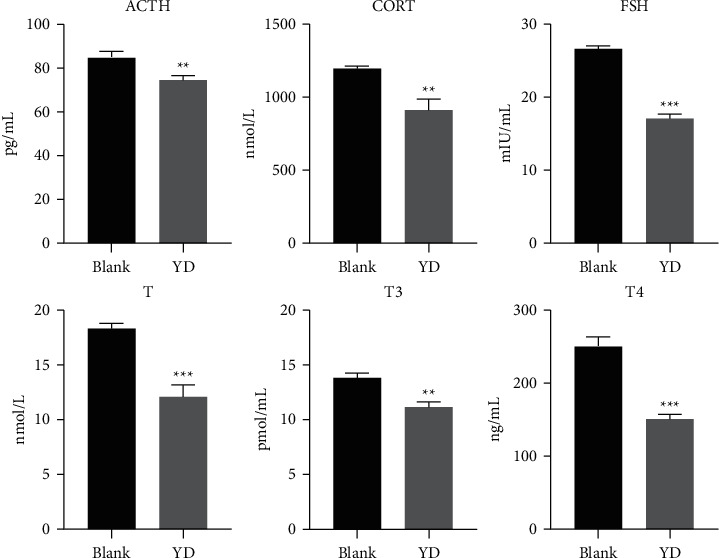
The biochemical characteristics used for the evaluation of YD. Bar plots represent the mean relative hormone intensities and standard deviations, and error bars represent the mean ± SD (Student's *t*-test: ^*∗*^significant difference from the control group at *p* < 0.05; ^*∗∗*^ significant difference from the control group at *p* < 0.01; ^*∗∗∗*^ significant difference from the control group at *p* < 0.001; compared with the blank group, the serum levels of T3, T4, ACTH, T, FSH, and CORT in the YD group were significantly decreased).

As shown in Figure 1, after receiving hydrocortisone, the weight of SD rats reduced considerably by the end of the 22nd day. Additionally, as seen in Figure 2, YD rats showed a number of behavioral and physiologic features, including weariness, decreased activity, a trend to cluster, an antipathy to cold, shivers, a decrease in appetite, and hair loss. Moreover, the biochemistry parameters of the blank and YD groups are generalized in Figure 3. Compared with the blank group, the serum levels of T3, T4, ACTH, T, FSH, and CORT in the YD group were considerably decreased. The hormones of the endocrine and urogenital systems were decreased in the YD group, which meant that the endocrine and urogenital systems were in a state of inhibition. Each of these findings revealed that a YD model had been successfully established. This corresponded to the findings of a previous study [16].

**Figure 4:**
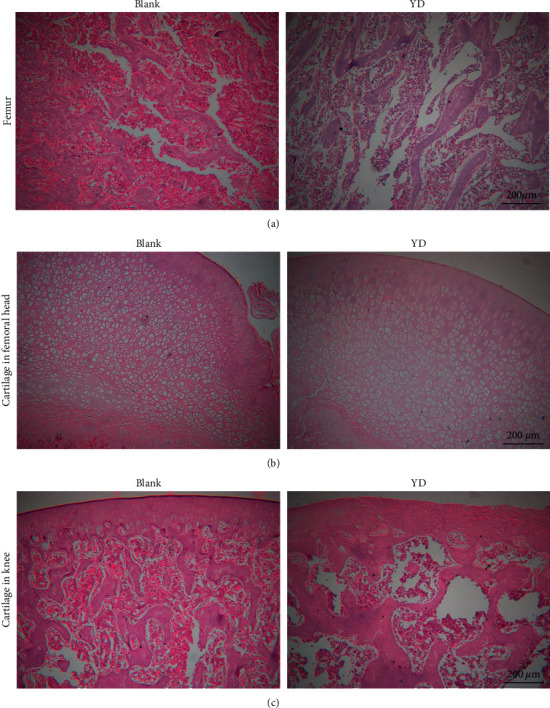
H&E staining of bone and cartilage tissue sections (a–c): magnification 100×.

**Figure 5:**
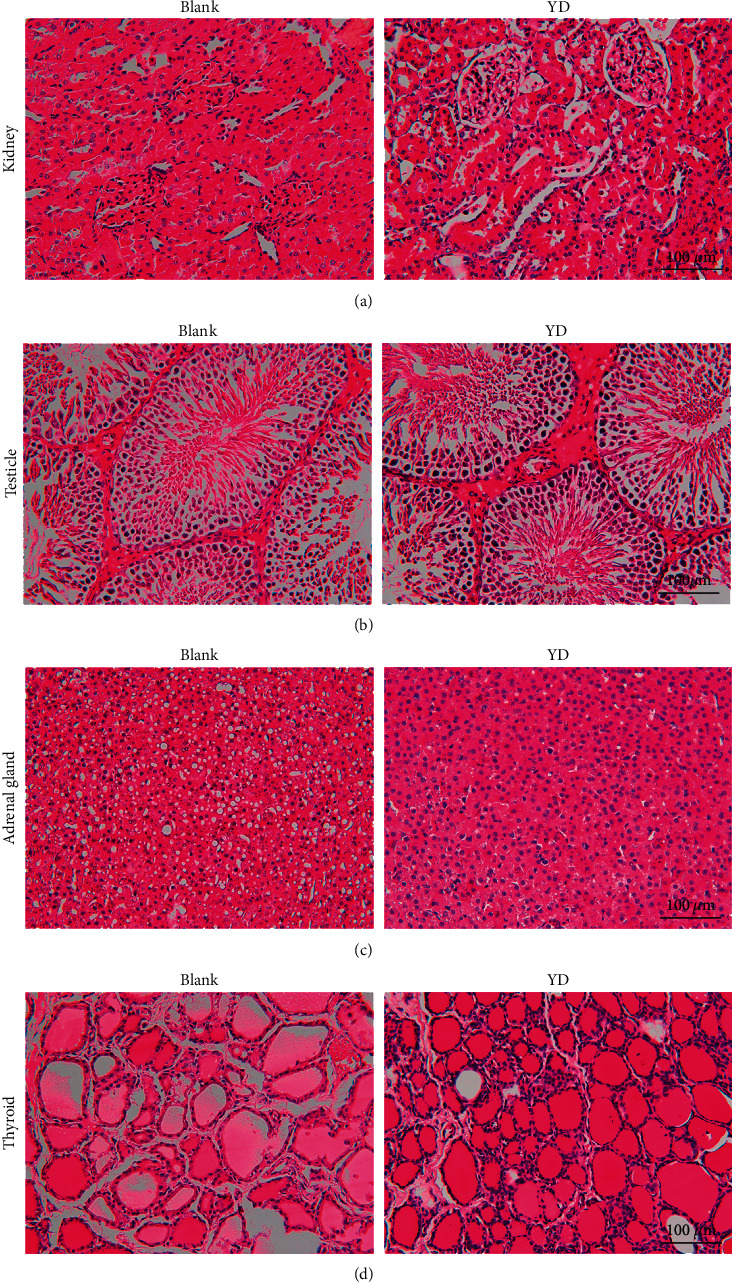
H&E staining of kidney, testes, adrenal gland, and thyroid tissue sections (a–d): magnification 400×.

To compare the histological differences between kidney-yang-deficient rats and normal rats, we performed H&E staining on bone, cartilage, kidney, testicle, thyroid, and adrenal gland sections from the normal and YD model groups. The results of H&E staining showed that the bones, cartilages, kidneys, testicles, and thyroid and adrenal glands had significant changes between the normal group and the YD group, as shown in Figures 4 and 5. As illustrated in Figure 4(a), in the blank group, a significant mass of femoral trabecular bone was densely distributed, and a large number of bone marrow hematopoietic cells were distributed between the bone trabecular bones. The trabecular bone was thick and strong, the trabecular bone spacing was small, and the trabecular bone distribution area accounted for a higher proportion of the observed field of view. In the YD group, the femoral shaft trabecula was loosely distributed and the number decreased, and a small number of bone marrow hematopoietic cells were distributed between these trabecular bones. The trabecular bone became thinner and elongated, and the local area was thicker. The trabecular bone spacing was larger, and the trabecular bone distribution area accounted for a lower proportion of the observed field of view. As illustrated in Figure 4(b), in the blank group, the cartilage in the femoral head had a large number of cartilage cells. The cells were densely distributed and tightly arranged. The cell body was large. The cartilage on the top of the cartilage in the femoral head was smooth and dense, and the cartilage matrix was more evenly distributed. In the YD group, the number of cartilage cells in the cartilage in the femoral head decreased, the cell distribution was looser, and the cell spacing was stretched. Moreover, the cartilage on the top of the cartilage in the femoral head was smooth but the density decreased, the distribution of the cartilage matrix decreased, and the density and elasticity of the cartilage in the overall cartilage in the femoral head decreased.

As illustrated in Figure 4(c), the cartilage in knees from the blank group was smooth and continuous, with dense chondrocytes and more cartilage matrix components in the cartilage layer. Additionally, the trabecular bones were densely distributed with a large number of bone marrow hematopoietic cells. The trabecular bone was thick and strong, the spacing is small, and the trabecular bone distribution area accounted for a higher proportion of the observed field of view. In the YD group, the cartilage in the knee lost its smooth and continuous characteristics, and the cartilage layer shrank and collapsed, with more fibrous components being present and only a small amount of cartilage cells and cartilage matrix components remaining. A small number of bone marrow hematopoietic cells were distributed between the trabeculae. The trabecular bone became thinner and elongated, and the local area was thicker. The trabecular bone spacing was larger, and the trabecular bone distribution area accounted for a lower proportion of the observed field of view.

As illustrated in Figure 5(a), there were no abnormal kidney renal tubular and glomerular changes in the blank group. Compared with the blank group, the renal glomerular of YD group rats expanded and the renal tubular dyeing became shallower, which indicated a change in glomerular filtration and reabsorption of renal tubules. These changes may be associated with the body's hormone levels, causing an increased level of glomerular filtration and renal tubular reabsorption function decline. As illustrated in Figure 5(b), intensive seminiferous tubule can be seen in the testicles of the blank group, in which all levels of spermatogenic cells and sperm components can be seen. Compared with the blank group, the spermatogenic tubules in the YD group were basically normal, and sperm formation could be seen, but the interstitial components of the seminiferous tubules increased, and the seminiferous tubule gaps widened. These changes may reduce the area of effective spermatogenesis in the testes to a certain extent. As illustrated in Figure 5(c), it can be seen that the zona fasciculata, the thickest part in the adrenal cortex, accounted for about 78% of the overall cortex and the oval nucleus in this part was larger. In the blank group, there was a lot of fat in the cytoplasm. As these lipid droplets were dissolved in regular slices, lots of shallow vacuolates can be seen in this area. The cells in the zona fasciculata secrete glucocorticoids, with cortisol and corticosterone accounting for the majority. In the YD group, the cytoplasm of the adrenal zona fasciculata cells was red and vacuolization rarely could be seen, which indicated they had fat deposition. The secretion function of zona fasciculata cells, regulated by adrenocorticotropic hormone secretion from adenohypophysis cells, is impacted by the increase of exogenous hormone level decrease. This was also confirmed from the low function cellular morphology. As illustrated in Figure 5(d), the thyroid gland was mainly composed of thyroid follicles. The section surface of thyroid follicles was larger in the blank group, and transparent colloids could be seen in these follicles. However, in the section surface of the YD group, the size of thyroid follicles decreased, and the number of follicles increased. The follicular epithelial cells shed and dropped into the colloid in local follicles. Due to thyroid atrophy, the volume of thyroid follicles was reduced, and the number of follicles in these sections was dense. Meanwhile, the colloids in these follicles were red, and the epithelial cells of these follicles were exfoliated and dropped with the colloid components in local follicles. Taken together, these findings indicated that injection with hydrocortisone resulted in changes in the cell structures of kidney, adrenal, thyroid, and testicular tissues. This again demonstrated that the hypothalamic-pituitary-thyroid axis, the hypothalamic-pituitary-gonadal axis, and the hypothalamic-pituitary-adrenal axis were inhibited in YD rats.

### 3.2. RNA-Seq, Transcriptome Assembly, and Function Notes

The transcriptomes of bones, cartilages, kidneys, testes, and thyroid and adrenal glands from normal rats and YD rats were separately sequenced using paired-end Illumina sequencing. All read sequences were deposited in the National Center for Biotechnology Information's (NCBI) Sequence Read Archive (SRA) database. The accession numbers were PRJNA734479, PRJNA734523, PRJNA73381, PRJNA743645, PRJNA743336, and PRJNA743922. Low-quality readings and adaptors were removed from the data set. Meanwhile, 45,819,034 and 44,183,040 clean reads were acquired from the bones of rats, which were the blank and YD samples, respectively, as shown in Table 1. The Q30 scores were found to be greater than 92% in the quality evaluation, and the percentages of GC were roughly 50%. Meanwhile，40,739,678 and 39,710,680 reads were mapped to the rat genome for the blank and YD bone samples, respectively. In all, 12,387 out of 15,811 (blank) and 12,143 out of 15,456 (YD) transcripts were annotated against the nonredundant (NR) NCBI protein database and Swiss-Prot database, respectively. Following the removal of low-quality reads and adaptor sequences, 44,146,126 and 41,999,440 clean reads were collected from the cartilages of rats, which were the blank or YD samples, respectively, as indicated in Table 1. Then, 39,772,734 and 37,773,322 reads were mapped to the rat genome for the blank and YD cartilage samples, respectively. In total, 13,052 out of 16,618 (blank) and 12,568 out of 16,053 (YD) transcripts were annotated against the above-mentioned databases, respectively. In the same way, 38,313,606 and 40,757,400 reads were mapped to the rat genome for the blank and YD kidney samples, respectively. In all, 12,813 out of 16,001 (blank) and 12,806 out of 16,063 (YD) transcripts were annotated against the above-mentioned databases, respectively. In the same way，37,626,116 and 41,017,358 reads were mapped to the rat genome for the blank and YD testicular samples, respectively. In total, 13,563 out of 17,880 (blank) and 13,507 out of 17,796 (YD) transcripts were annotated against the aforementioned databases, respectively. The above data are summarized in Table 1. The relevant data of adrenal gland and thyroid samples are also summarized in Table 1.

### 3.3. Comparative Investigation of Differentially Expressed Genes

Differential expression analysis of the blank and YD bone samples showed 750 genes that were prominently differentially expressed between the YD and blank groups (log_2_ fold change ≥1 or ≤−1 and *p* ≤ 0.001), including 117 upregulated genes and 633 downregulated genes (YD versus blank). For the cartilage samples from blank and YD groups, contrasting expression analysis identified 3128 genes that were prominently differentially expressed between the YD and blank groups (log_2_ fold change ≥1 or ≤−1 and *p* ≤ 0.001), comprising 1200 genes that were upregulated and 1928 genes that were downregulated (YD versus blank). Differential expression analysis of the blank and YD kidney samples showed 716 genes that were significantly differentially expressed between the YD and blank groups (log_2_ fold change ≥1 or ≤−1 and *p* ≤ 0.001), including 450 upregulated genes and 266 downregulated genes (YD versus blank). With regard to the samples of blank and YD tests, differential expression analysis identified 123 genes that were prominently differentially conveyed between the YD and blank groups (log_2_ fold change ≥1 or ≤−1 and *p* ≤ 0.001), including 45 upregulated genes and 78 downregulated genes (YD versus blank). The above data are shown in Table 2. The relevant data of adrenal gland and thyroid samples are also summarized in Table 2.

### 3.4. GO and KEGG Enrichment Analysis of DEGs in Bone, Cartilage, Kidney, Testicle, Thyroid, and Adrenal Gland with YD

**Figure 6:**
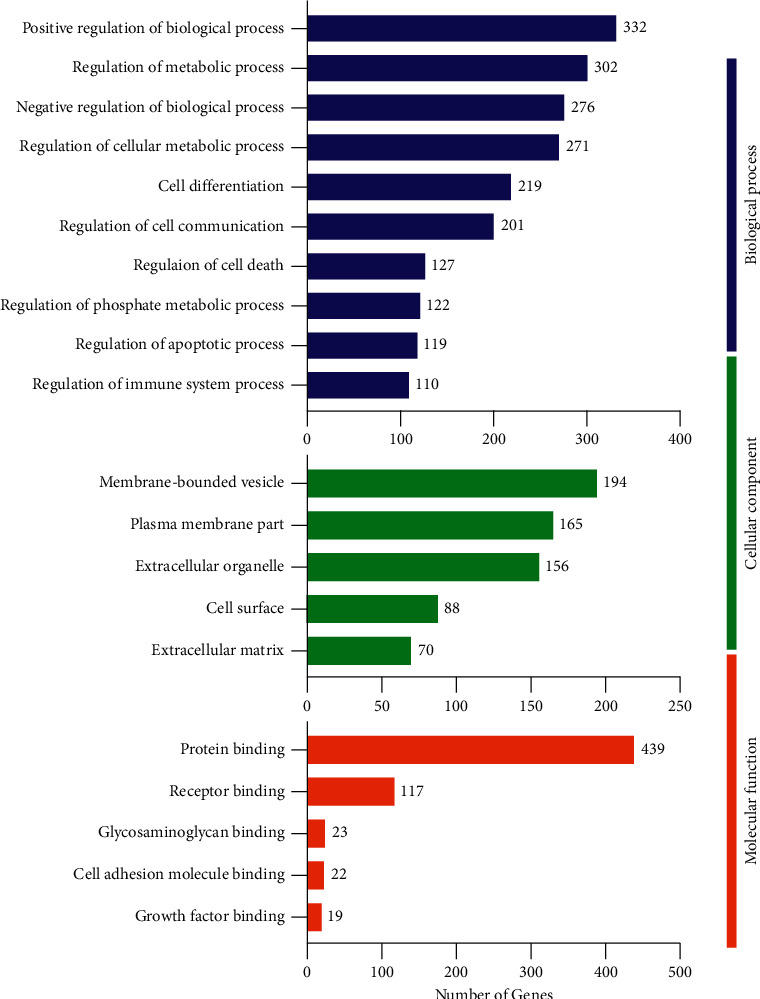
GO enrichment analysis of differentially expressed genes in bone under YD. Histogram display of GO enrichment analysis of DEGs. 2e results are divided into three categories: cellular components, molecular functions, and biological processes. The 2e *x*-axis represents the number of DEGs corresponding to each GO term, and the *y*-axis represents the name of each GO term.

**Figure 7:**
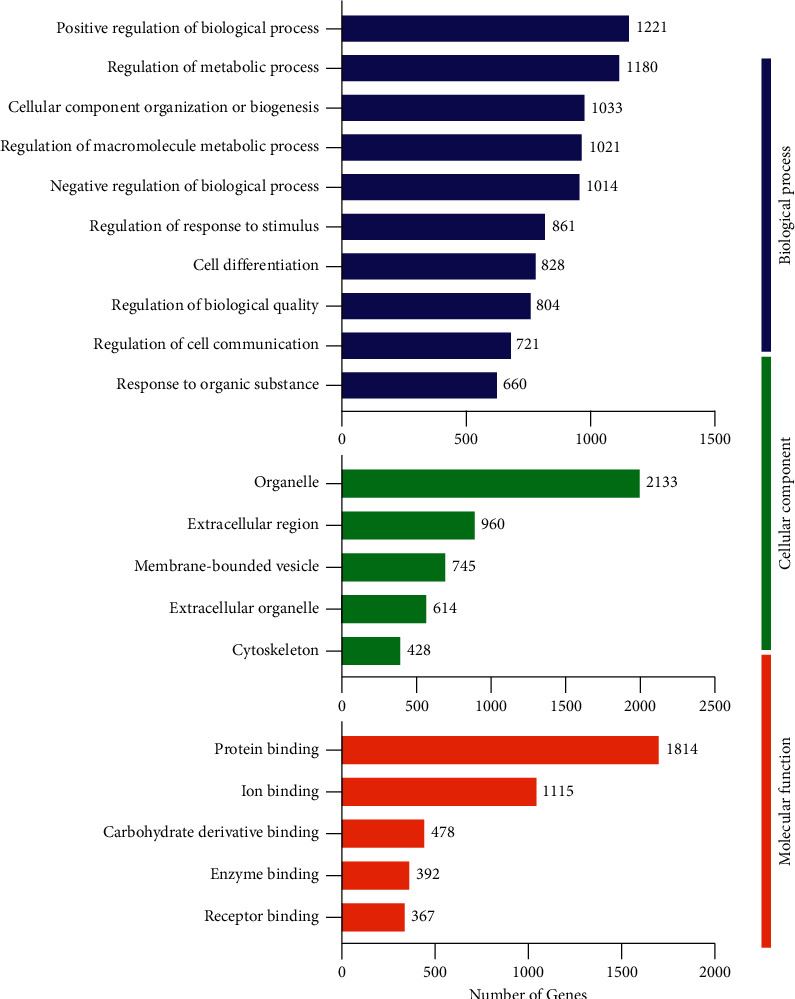
GO enrichment analysis of differentially expressed genes in cartilage under YD. Histogram display of GO enrichment analysis of DEGs. 2e results are divided into three categories: cellular components, molecular functions, and biological processes. The 2e *x*-axis represents the number of DEGs corresponding to each GO term, and the *y*-axis represents the name of each GO term.

We performed GO enrichment studies in order to get insight into the DEGs implicated in the bone, cartilage, kidney, testis, adrenal gland, and thyroid under the condition of YD. As illustrated in Figure 6, the significantly enriched GO terms associated with biological processes in the bone included regulation of biological processes, regulation of metabolic processes, regulation of cellular metabolic processes, cell differentiation, regulation of cell communication, regulation of the phosphate metabolic process, regulation of the apoptotic process, regulation of cell death, and regulation of immune system processes. The obviously enriched GO terms relevant to cellular components mainly involved membrane-bounded vesicle, plasma membrane part, extracellular organelle, and cell surface and extracellular matrix. The obviously enriched GO terms relevant to molecular function mainly involved protein binding, receptor binding, glycosaminoglycan binding, cell adhesion molecule binding, and growth factor binding. As illustrated in Figure 7, the considerably enriched GO keywords for the cartilage are primarily related to biological processes, mainly regulation of biological processes, regulation of metabolic processes, cellular component organization or biogenesis, regulation of response to stimulus, cell differentiation, regulation of biological quality, and regulation of cell communication. Significantly enriched GO keywords associated with cellular components were mostly concerned with organelle, extracellular region, membrane-bounded vesicle, and cytoskeleton. The significantly enriched GO terms related to molecular function mainly involved protein binding, enzyme binding, ion binding, carbohydrate derivative binding, and receptor binding. As illustrated in Supplementary Figure 1, for the kidney, the obviously enriched GO terms related to biological processes mainly involved regulation of biological processes, cell differentiation, regulation of biological quality, cellular developmental process, regulation of cell communication, regulation of signal transduction, regulation of the phosphate metabolic process, regulation of the apoptotic process, and regulation of the immune system process. The obviously enriched GO terms relevant to cellular components mostly involved extracellular region, membrane-bounded vesicle, plasma membrane part, cell surface, and extracellular matrix. The obviously enriched GO terms relevant to molecular function mostly involved protein binding, ion binding, receptor binding, cytokine receptor binding, and carbohydrate binding. As illustrated in Supplementary Figure 2, for the testes, the obviously enriched GO terms relevant to biological processes mostly involved regulation of protein phosphorylation, positive regulation of cell death, response to steroid hormone, regulation of cell adhesion, negative regulation of immune system process, regulation of T cell differentiation, and positive regulation of the apoptotic signaling pathway. The obviously enriched GO terms relevant to cellular components mostly involved extracellular space, membrane microdomain, chromosome, centromeric region, and integral component of organelle membrane and cytosolic part. The obviously enriched GO terms related to molecular function mostly involved transition metal ion binding, enzyme activator activity, iron ion binding, extracellular matrix structural constituent, and protein kinase regulator activity. In the same way as above, the relevant data of adrenal gland and thyroid samples are summarized in Supplementary Figures 3 and 4.

**Figure 8:**
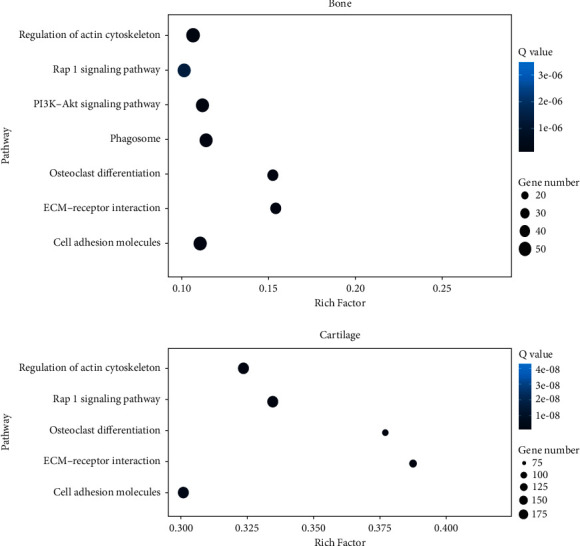
Scatter plot display of KEGG enrichment analysis of DEGs. The 2e *x*-axis represents each enrichment factor, representing the proportion of DEGs involved in each KEGG pathway among all identified DEGs, and the *y*-axis represents each enrichment pathway. The 2e size of the dot reflects the number of DEGs, and the color of the dot reflects the adjusted *p* value (Q value).

KEGG pathway enrichment analyses were used to further investigate the physiological processes and pathways underlying these differentially expressed genes implicated in bone, cartilage, kidney, testis, adrenal gland, and thyroid function in response to YD. As illustrated in Figure 8, the obviously enriched pathways in the bone primarily involved actin cytoskeleton regulation, the Rap1 signaling route, the PI3K-Akt signaling pathway, and osteoclast differentiation, ECM-receptor interaction, and cell adhesion molecules. Furthermore, as depicted in Figure 8 for the cartilage, the obviously enriched pathways mainly involved regulation of actin cytoskeleton, ECM-receptor interaction, Rap1 signaling pathway, osteoclast differentiation, and cell adhesion molecules. As illustrated in Supplementary Figure 5, for the kidney, the obviously enriched pathways mainly involved osteoclast differentiation, primary immunodeficiency, PI3-Akt signaling pathway, leukocyte transendothelial migration, ECM-receptor interaction, complement and coagulation cascades, and cell adhesion molecules (CAMs). Furthermore, as depicted in Supplementary Figure 6 for the testicle, the obviously enriched pathways mainly involved cellular senescence, T cell receptor signaling pathway, PPAR signaling pathway, primary immunodeficiency, cell adhesion molecules, and autoimmune thyroid disease. In the same way as above, the relevant data of adrenal gland and thyroid samples are summarized in Supplementary Figures 7 and 8.

### 3.5. YD Decreases the Expression Levels of Bone Cell Markers and Bone Homeostasis Markers

In clinical practice, we find that YD is very common in the elderly. Modern researchers have indicated that YD is mostly caused by functional abnormalities of varying degrees in the hypothalamic-pituitary-target gland axis (adrenal, thyroid, and gonad), and it is characterized by cold limbs, limited movement, sluggish responsiveness, decreased hunger, and cowering, among other characteristics [4, 17]. This is actually a state of inhibition of human function. The elderly with kidney-yang deficiency often have bone-related diseases. YD has a deleterious influence on the skeleton as a pathological condition, specifically as it is a disorder of bone homeostasis. Therefore, we postulated that YD would decrease bone development by inhibiting bone cell markers involved in bone formation. We thus tested some markers related to bone cells. As revealed in Table 3, the expression levels of osteoblast and osteocyte markers were dramatically decreased in the bone under the influence of YD, such as *Col1a1*, *Ibsp*, *Bglap*, *Omd*, *Dcn*, *Dmp1*, *Sost*, *Phex*, and *Mepe*. Meanwhile, we evaluated the expression levels of a number of local genes that positively regulate bone homeostasis. As revealed in Table 3, the expression levels of *Pth1r*, *Bmp3*, *Bmp8a*, *Fgfr3*, and *Sox4* were significantly decreased under YD.

### 3.6. YD Reduces Chondrocyte Proliferation by Inhibiting Chondrocyte Proliferation and Promoting Chondrocyte Differentiation

According to our RNA-Seq data, the expression levels of *Sox9*, *Sox8*, *Sox5*, *Sox4*, *Acan*, *Col2a1*, *Col9a1*, *Col11a1*, *Hapln1*, and *Wwp2* were decreased in response to YD. These genes were pancartilaginous early chondrocyte markers. Additionally, YD lowered the expression of proliferative and prehypertrophic chondrocyte markers such as *Fgfr3*, *Matn1*, *Comp*, and *Runx2*. Moreover, the expression levels of major prehypertrophic and hypertrophic chondrocyte markers including *Pth1r*, *Sp7*, *Ihh*, *Bmp6*, and *Ibsp* decreased under the influence of YD, as shown in Table 4.

### 3.7. YD Impacts the Expression Levels of Kidney and Testicle Markers Involved in Kidney and Testicle Function

As indicated in Table 5, YD had an effect on the expression levels of renal and testicle markers involved in kidney and testicular function. According to our RNA-Seq data, the expression levels of *Cd28*, *Slc4a5*, *Slc38a3*, *Slc22a2*, *Slc22a12*, *Aqp7*, and *Ephb1* were slightly decreased in response to YD. Furthermore, the expression levels of *Hmgb2*, *Col1a1*, *Cyp2a1*, *Ddit4*, *Col3a1*, *Igfbp4*, *Ccdc39*, *C1galt1*, and *Scrn1* were slightly decreased in response to YD.

### 3.8. The Effect of YD on the Expression Levels of Genes with Consistent Patterns in Both the Adrenal Gland and Bone (or Cartilage)

In order to find the connection of the adrenal gland with bone and cartilage, we analyzed the DEGs with accordant patterns in both the adrenal gland and bone, and the DEGs with consistent patterns in both the adrenal gland and cartilage. As indicated in Table 6, the expression levels of 6 genes were clearly raised in both the adrenal gland and bone, such as *Alox15*, *Fam111a*, *Rsad2*, *Asns*, *Creld2*, and *Ddit3*. The expression levels of 5 genes were clearly decreased in both the adrenal gland and bone, including *Hhex*, *Myo10*, *Adamts9*, *Prkce*, and *Thrsp*. As indicated in Supplementary Table 1, the expression levels of 6 genes were clearly raised in both the adrenal gland and cartilage, such as *S100a9*, *S100a8*, *Car2*, *Cyba*, *Alox15*, and *Mmp8*. The expression levels of 8 genes were clearly decreased in both the adrenal gland and cartilage, including *Insig1*, *Nr1d1*, *Slc26a2*, *Igf1*, *Ppard*, *Adamts9*, *Plat*, and *Lama5*.

### 3.9. The Effect of YD on the Expression Levels of Genes with Consistent Patterns in Both the Thyroid and Bone (or Cartilage)

In order to find the connection of the thyroid with bone and cartilage, we analyzed the DEGs with accordant patterns in both the thyroid and bone and the DEGs with consistent patterns in both the thyroid and cartilage. As illustrated in Supplementary Table 2, 6 genes' expression levels were considerably raised in both the thyroid and bone, including *Alox15*, *Rsad2*, *Bcl3*, *Grem1*, *Cytl1*, and *Hapln1*. The expression levels of 3 genes were clearly decreased in both the thyroid and bone, such as *Col1a1*, *Col1a2*, and *Col3a1*. As shown in Supplementary Table 3, the expression levels of 5 genes were clearly increased in both the thyroid and cartilage, including *S100a9*, *S100a8*, *Cytl1*, *Slpi*, and *Pla2g2a*. The expression levels of 5 genes were significantly decreased in both the thyroid and cartilage, such as *Col1a1*, *Col1a2*, *Col3a1*, *Col15a1m*, and *Sfrp2*.

### 3.10. The Results of DEGs Verified by qRT-PCR Were Consistent with RNA-Seq

**Figure 9:**
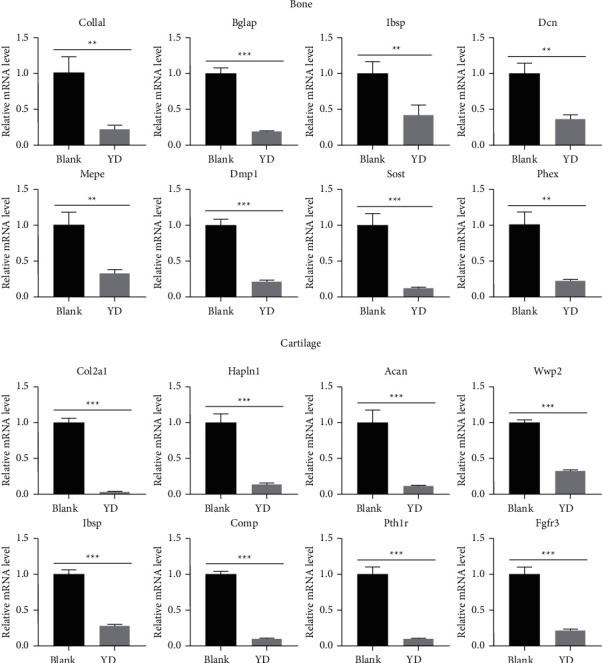
Gene expression levels of differentially expressed genes validated by qRT-PCR. Data are presented as the mean with standard deviation from technical triplicates for each experiment representative of several independent experiments. ^*∗∗*^ represents *p* < 0.01 and ^*∗∗∗*^ represents *p* < 0.001 in a *t*-test for the difference in gene expression levels. Gene expression levels for individual genes are presented as the fold change between the YD group and the blank group.

Following that, we used qRT-PCR to confirm the correctness and reliability of our RNA-Seq results for 16 DEGs, including 8 downregulated DGEs (*Col1a1*, *Ibsp*, *Bglap*, *Dcn*, *Dmp1*, *Sost*, *Phex*, and *Mepe*) involved in osteoblasts and serving as osteocyte markers and 8 downregulated DEGs (*Col2a1*, *Acan*, *Hapln1*, *Wwp2*, *Ibsp*, *Comp*, *Fgfr3*, and *Pth1r*) involved as chondroprogenitor markers and growth plate chondrocyte markers. Supplementary Table 4 contains the forward and reverse primer sequences for qRT-PCR. Each gene's relative fold change in expression was standardized against the internal control gene *Gapdh*. As illustrated in Figure 9, the expression patterns of these 16 genes were consistent with our RNA-Seq research results.

## 4. Discussion

“Kidney governing bones” is a fundamental theory in TCM in which is used as the gold standard for the treatment of bone diseases. The kidney is responsible for the growth, development, and repair of the skeleton. However, the precise molecular mechanism underlying “kidney governing bones” is still not clearly understood. Kidney-yang deficiency is a typical pattern of syndrome in TCM. The hypothalamus-pituitary-target gland (adrenal, thyroid, and gonad) axis has been identified as the primary mechanism of YD incidence, with variable degrees of functional abnormalities [3, 4]. When the kidney is in a pathological state, the bones may undergo corresponding changes. As is well known, the majority of complex diseases are caused by disruption of biological networks as a result of the malfunctioning of numerous genes or their products. In this study, we performed RNA-Seq to analyze the gene expression patterns of bone, cartilage, kidney, testicle, thyroid, and adrenal gland in Sprague Dawley (SD) rats (kidney-yang-deficient rats and normal rats). After the comprehensive analysis of the sequencing results, we attempted to find the exact molecular mechanism of “kidney governing bones.” Our differential expression analysis of the blank and YD bone samples showed 750 genes that were obviously differently expressed across the two groups, including 117 upregulated genes and 633 downregulated genes (YD versus blank). The differential expression analysis of the blank and YD cartilage samples identified 3128 genes that were obviously differently expressed between the YD and blank groups, including 1200 upregulated genes and 1928 downregulated genes (YD versus blank). Furthermore, the kidney, testicle, thyroid, and adrenal gland all had different numbers of obviously upregulated genes and obviously downregulated genes. According to GO enrichment analysis, the bone transcriptome's significantly enriched GO terms included protein binding, receptor binding, glycosaminoglycan binding, cell adhesion molecule binding, and growth factor binding. The cartilage transcriptome's strongly enriched GO keywords included protein binding, ion binding, carbohydrate derivative binding, enzyme binding, and receptor binding. The significantly enriched GO terms for the kidney, testicle, adrenal gland, and thyroid transcriptome mainly involved protein binding, ion binding, receptor binding, macromolecular complex binding, cytoskeletal protein binding, cytoskeletal protein binding, enzyme inhibitor activity, and extracellular matrix structural constituent and protein kinase regulator activity.

Additionally, we investigated the bone indicators associated with bone homeostasis. In YD rats, osteoblast and osteocyte marker expression levels were dramatically lowered in the bone. These findings indicated that YD could be able to restrict bone production by reducing bone cell markers involved in bone formation. Meanwhile, it disrupts bone homeostasis. Notably, the levels of osteoblast and osteocyte markers were considerably lowered in the bone under the influence of YD, such as *Col1a1*, *Ibsp*, *Bglap*, *Omd*, *Dcn*, *Dmp1*, *Sost*, *Phex*, and *Mepe*. Multiple local genes that positively regulate bone homeostasis, such as *Pth1r*, *Bmp3*, *Bmp8a*, *Fgfr3*, and *Sox4*, had their expression levels drastically lowered by the influence of YD. Sost is a circulating protein that has the ability to decrease bone growth by inhibiting the Wnt/*β*-catenin signaling pathway [18]. In the kidney and bone, Pth1r plays a role in controlling systemic mineral ion homeostasis by maintaining calcium-phosphate balance via crosstalk between the bone and kidney [19]. Bmp3 is a member of the transforming growth factor beta superfamily, which is important in embryonic and postnatal bone development [20]. Fgfr3, a fibroblast growth factor receptor, regulates endochondral ossification and bone development [21]. Furthermore, we analyzed several cartilage markers. We found that YD could reduce chondrocyte proliferation by inhibiting chondrocyte proliferation and promoting chondrocyte differentiation. Sox8 is abundantly expressed in many tumor cells [22]. Sox9 is required for cartilage formation. It is a master transcription factor that regulates target genes like *Sox8*, *Sox5*, *Sox4*, *Acan*, *Col2a1*, *Col9a1*, *Col11a1*, *Hapln1*, and *Wwp2* [23–25]. We then examined the levels of expression of growth plate cartilage markers at various stages of differentiation. Fgfr3, Matn1, Comp, and Runx2 were shown to have lower expression levels in response to YD. These markers were found to be associated with proliferative and prehypertrophic chondrocytes. As a result of the influence of YD, the expression levels of several significant markers of prehypertrophic and hypertrophic chondrocytes were lowered. These markers included Pth1r, Sp7, Ihh, Bmp6, and Ibsp, which are all associated with hypertrophic chondrocytes. As a result, these outcomes were accordant with the above findings that the effect of YD could inhibit bone formation through suppressing bone cell markers that were involved in bone formation and YD could inhibit cartilage function by inhibiting chondrocyte proliferation and promoting chondrocyte differentiation. Thus, we found that, in the state of kidney-yang deficiency, the function of bone and cartilage was also inhibited.

Previously, our group explored the molecular mechanisms related to bone development, growth, and regeneration using modern biology technologies, such as RNA-Seq and ITRAQ, and demonstrated that the underlying molecular mechanisms were highly consistent with the “kidney governing bones” theory. The results of this study coincide with our previous studies [6–10]. We also looked at markers for renal and testis function. YD reduced the expression of certain kidney and testis markers related in kidney and testis function. Slc22a12 and Slc22a2 are two different forms of solute carrier (Slc) family 22 transporters that allow the transport of a variety of substrates through biological membranes [26]. Hmgb2 is particularly common in the testis. Hmgb2−/− males have decreased fertility, which is associated with germ cell loss, spermatid abnormalities, and spermatozoa immobility [27]. During spermatogenesis, Col1a1 regulates the adherence of spermatogonia and preleptotene spermatocytes to the basement membrane, as well as their dissociation and migration into the lumen [28]. Ddit4 is mitochondrial-localized and is required for the reduction of ROS generation and release by mitochondria [29]. Ddit4 expression is critical for cell survival during germ cell development, as only cells with a high level of expression appear to be adequately protected against oxidative stress [30]. CYP2A1 is closely related to testicular function. Reduced levels of CYP2A1 protein may result in impaired testicular estradiol and testosterone metabolism. It has been shown to have an effect on the testis and male fertility [31]. Therefore, these results were consistent with the above findings. These results suggested that, in the state of kidney-yang deficiency, the functions of the kidney and testis were inhibited to some extent.

Studies have shown that the mechanism of occurrence for YD is the hypothalamus-pituitary-adrenal gland axis of functional disorders, and in the state of kidney-yang deficiency, the function of the adrenal gland is inhibited [32]. In previous results, we found that the function of bone and cartilage was inhibited. In order to find the connection between adrenal gland and bone and cartilage, we analyzed the DEGs with consistent patterns in both the adrenal gland and bone, and the DEGs with consistent patterns in both the adrenal gland and cartilage. Our results showed that some genes positively regulating the function of bone and cartilage were downregulated, while some genes negatively regulating bone and cartilage were upregulated. It has been discovered that the arachidonate 15-lipoxygenase (*Alox15*) gene acts as a negative regulator of peak BMD in mice [33]. Alox15-knockout mice had higher BMD than normal mice, and Alox15 inhibitors enhance the BMD and bone loss rate in a rat osteoporosis model [33]. Ddit3 is a transcription factor that plays a critical role in ER stress, a process that occurs during cell apoptosis [32]. Myo10 has been shown to be involved in the regulation of osteoclast (OC) adhesion, podosome location, and differentiation. In mice lacking Myo10 activity, trabecular bone mass diminishes, which appears to be a significant part to accelerated bone resorption and OC formation [34]. Increased expression of Alox15 and Ddit3 and decreased expression of Myo10 impairs bone function. S100 proteins are intracellular calcium-binding proteins (9–14 kDa). It has the ability to regulate critical biological pathways, including regulating the cytoskeleton [35], cell migration and adhesion [36], and host oxidative defense [37, 38]. S100A8 and S100A9 are present intracellularly in granulocytes, monocytes, and macrophages in the early stages of differentiation [39, 40]. Due to its effect on cytokine generation, extracellular S100A8 is considered a proinflammatory molecule [41]. Additionally, it has been demonstrated that chondrocytes express S100A8 and S100A9 [42]. Slc26a2 is a ubiquitously expressed sulfate transporter on the cell membrane that allows intracellular sulfate delivery [43, 44]. This gene has been linked to approximately 5 hereditary skeletal illnesses, including achondrogenesis type IB (ACG1B), diatrophic dysplasia (DTD), recessive multiple epiphyseal dysplasia (rMED), atelosteogenesis type II (AO2), and dysplastic spondylolysis [45–47]. Therefore, these results were consistent with the above findings. These results also suggested that, in the state of kidney-yang deficiency, the function of the bone, cartilage, and adrenal gland were inhibited to some extent. Moreover, these organizations were closely related to each other.

Meanwhile, in the state of kidney-yang deficiency, the function of the thyroid is also inhibited [32]. In previous results, we found that the function of bone and cartilage was inhibited. In order to find the connection of the thyroid with them, we analyzed the DEGs with consistent patterns in both the thyroid and bone, and the DEGs with consistent patterns in both the thyroid and cartilage. Our results showed that some genes positively regulating the function of bone and cartilage were downregulated, while some genes negatively regulating the bone and cartilage were upregulated. Our study demonstrates that BCL3 interacts with TRAF6 via its ankyrin-repeat domain and suppresses osteoclastogenesis in macrophages generated from bone marrow. TRAF6 increases RANKL-induced osteoclastogenesis via unique noncanonical NF-*κ*B signaling mediated by BCL3 deubiquitination. This implies that BCL3 is an important modulator of bone loss-related disorders [48]. Osteogenesis imperfecta was formerly thought to be a dominantly inherited disease produced by mutations in either of the type I collagen genes (*COL1A1* and *COL1A2*) [49]. Calprotectin (S100A8/A9) is a damage-associated molecular pattern (DAMP) that is abundant in seronegative arthritis [50–53]. These results were consistent with the above findings. They also suggest that, in the state of kidney-yang deficiency, the function of the bone, cartilage, and thyroid were inhibited to some extent. Meanwhile these organizations are closely related to each other.

## 5. Conclusion

**Figure 10:**
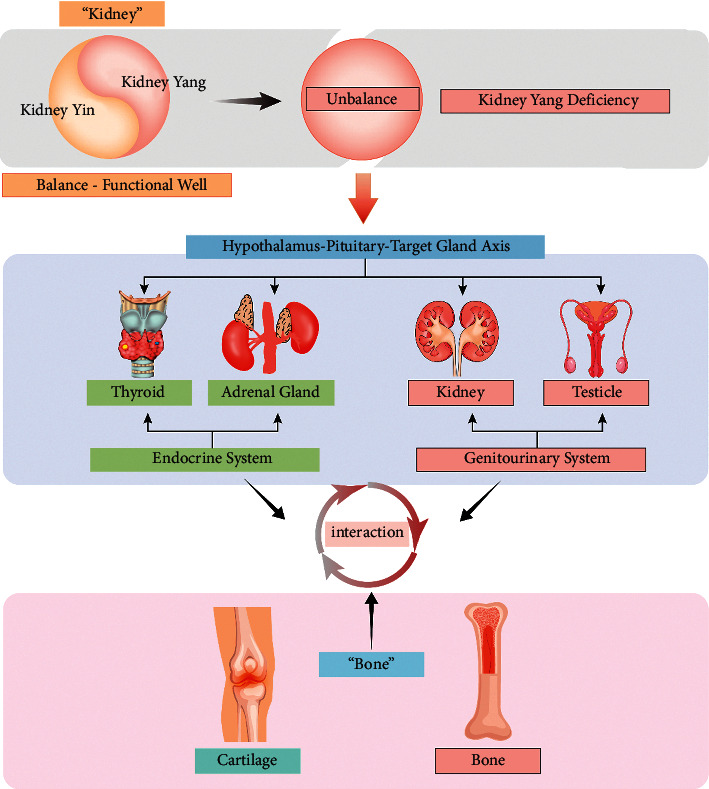
The illustration of the “kidney governing bones” theory. “Kidney” and “bone” in the illustration represent kidney and bone in traditional Chinese medicine. Kidney and bone in the illustration represent the kidney and bone in anatomical concept.

In summary, our work established that YD may impede bone production by suppressing bone cell markers involved in bone formation. Meanwhile, YD could inhibit cartilage function by inhibiting chondrocyte proliferation and promoting chondrocyte differentiation. In the state of kidney-yang deficiency, the function of bone and cartilage was inhibited. We found that “kidney governing bones” was not caused by the change of a single gene but rather the change of a biological network caused by the functional change of many genes or their products. As we all know, the kidneys, testes, and thyroid and adrenal glands are involved in the functions of the genitourinary and endocrine systems. This change in this biological network may be the result of the coordinated regulation of the endocrine urogenital system. This also coincided with the holistic view of TCM that the human body itself is an organic whole and the functional activities of each organ coordinate with each other and affect each other pathologically. The illustration of the “kidney governing bones” theory is provided in Figure 10.

## Figures and Tables

**Table 1:** Statistics summary of transcriptome sequencing and assembly (YD vs. blank).

Statistics	Bone-blank	Bone-YD	Cartilage-blank	Cartilage-YD	Kidney-blank	Kidney-YD	Testis-blank	Testis-YD	Adrenal gland-blank	Adrenal gland-YD	Thyroid-blank	Thyroid-YD
Clean reads	45,819,034	44,183,040	44,146,126	41,999,440	42,782,316	45,000,646	41,258,124	44,960,296	38,910,682	42,737,208	44,242,664	45,370,832
Q30 percentage	92.74	93.07	92.88	93.15	92.31	92.80	93.44	93.58	93.67	93.61	94.90	94.89
GC percentage	50.93	51.19	51.47	50.77	49.79	49.44	52.54	53.11	50.88	51.76	51.01	50.91
Total mapped reads	40,739,678	39,710,680	39,772,734	37,773,322	38,313,606	40,757,400	37,626,116	41,017,358	35,278,958	38,539,194	40,703,312	41,711,728
Total transcripts	15,811	15,456	16,618	16,053	16,001	16,063	17,880	17,796	15,709	15,895	15,878	15,835
Known transcripts	12,387	12,143	13,052	12,568	12,813	12,806	13,563	13,507	12,651	12,850	13,368	13,276

**Table 2:** Statistic overview of DEGs (YD vs. blank).

Statistics (number)	Bone	Cartilage	Kidney	Testicle	Adrenal Gland	Thyroid
Differentially expressed genes	750	3,128	716	123	1,367	459
Upregulated genes	117	1,200	450	45	1,224	363
Downregulated genes	633	1,928	266	78	143	96

**Table 3:** The expression levels of bone cell markers and bone homeostasis markers (YD vs. blank).

Gene name	Expression level (FPKM)		log_2_ fold change (YD/blank)	*p* value
	Blank	YD		
*Osteoblast markers*				
Collagen type I alpha 1 chain (*Col1a1*)	501.94	132.60	−1.92	≤0.001
Bone gamma-carboxyglutamate protein (*Bglap*)	570.37	86.95	−2.71	2.88*E* − 228
Integrin-binding sialoprotein (*Ibsp*)	102.49	47.45	−1.11	8.92*E* − 72
Decorin (*Dcn*)	57.61	25.88	−1.15	3.05*E* − 35
Osteomodulin (*Omd*)	15.61	1.82	−3.10	2.38*E* − 40

*Osteocyte markers*				
Matrix extracellular phosphoglycoprotein (*Mepe*)	75.94	21.92	−1.79	9.99*E* − 91
Dentin matrix acidic phosphoprotein 1 (*Dmp1*)	54.38	15.75	−1.79	4.15*E* − 109
Sclerostin (*Sost*)	31.92	6.32	−2.34	5.40*E* − 19
Phosphate regulating endopeptidase homolog, X-linked (*Phex*)	3.30	0.67	−2.30	2.16*E* − 16

*Bone homeostasis markers*				
Parathyroid hormone 1 receptor (*Pth1r*)	12.89	4.12	−1.65	7.70*E* − 24
SRY box 4 (*Sox4*)	9.26	3.30	−1.49	2.67*E* − 27
Bone morphogenetic protein 8a (*Bmp8a*)	2.36	0.89	−1.41	1.05*E* − 07
Fibroblast growth factor receptor 3 (*Fgfr3*)	1.85	0.66	−1.49	4.79*E* − 05
Bone morphogenetic protein 3 (*Bmp3*)	1.61	0.31	−2.38	1.25*E* − 08

**Table 4:** Gene expression levels of chondroprogenitor markers and growth plate chondrocyte markers (YD vs. blank).

Gene name	Expression level (FPKM)		log_2_ fold change (YD/blank)	*p* value
	Blank	YD		
*Chondroprogenitor markers*				
Collagen type II alpha 1 chain (*Col2a1*)	8426.30	632.19	−3.74	≤0.001
Hyaluronan and proteoglycan link protein 1 (*Hapln1*)	570.05	166.55	−1.78	≤0.001
Aggrecan (*Acan*)	454.77	155.12	−1.56	≤ 0.001
WW domain containing E3 ubiquitin protein ligase 2 (*Wwp2*)	125.40	62.16	−1.01	2.82*E* − 123
Collagen type XI alpha 1 chain (*Col11a1*)	231.33	36.81	−2.65	≤0.001
Collagen type IX alpha 1 chain (*Col9a1*)	313.91	28.55	−3.46	≤0.001
SRY box 9 (*Sox9*)	38.73	12.05	−1.68	2.16*E* − 104
SRY box 4 (*Sox4*)	27.88	5.72	−2.29	9.67*E* − 132
SRY box 5 (*Sox5*)	6.85	2.37	−1.53	7.65*E* − 26
SRY box 8 (*Sox8*)	2.85	1.28	−1.15	8.53*E* − 05

*Growth plate chondrocyte markers*				
Integrin-binding sialoprotein (*Ibsp*)	1529.00	738.60	−1.05	≤0.001
Cartilage oligomeric matrix protein (*Comp*)	383.11	79.06	−2.28	≤0.001
Parathyroid hormone 1 receptor (*Pth1r*)	242.59	47.21	−2.36	≤0.001
Fibroblast growth factor receptor 3 (*Fgfr3*)	59.14	19.98	−1.57	6.76*E* − 121
Runt-related transcription factor 2 (*Runx2*)	25.66	11.43	−1.17	2.68*E* − 57
Bone morphogenetic protein 6 (*Bmp6*)	22.08	8.71	−1.34	3.17*E* − 16
Indian hedgehog protein (*Ihh*)	23.45	8.61	−1.45	9.67*E* − 29
Transcription factor Sp7 (Sp7)	46.28	8.20	−2.50	4.95*E* − 128
Matrilin 1, cartilage matrix protein (*Matn1*)	8.40	3.77	−1.16	3.88*E* − 07

**Table 5:** The expression levels of kidney and testicle markers involved in kidney and testicle function (YD vs. blank).

Gene name	Expression level (FPKM)		log_2_ fold change (YD/blank)	*p* value
	Blank	YD		
*Kidney markers*				
Solute carrier family 22 member 2 (*Slc22a2*)	316.76	153.09	−1.05	2.38*E* − 221
Solute carrier family 22 member 12 (*Slc22a12*)	246.90	119.68	−1.04	1.57*E* − 174
Aquaporin 7 (Aqp7)	98.41	48.27	−1.03	5.30*E* − 44
Solute carrier family 38, member 3 (*Slc38a3*)	124.91	47.03	−1.41	5.03*E* − 154
Solute carrier family 4 member 5 (*Slc4a5*)	3.00	1.08	−1.47	7.70*E* − 10
Eph receptor B1 (*Ephb1*)	1.19	0.23	−2.34	1.49*E* − 07
Cd28 molecule (*Cd28*)	2.94	0.12	−4.61	1.01*E* − 28

*Testis markers*				
High mobility group box 2 (*Hmgb2*)	134.60	37.49	−1.84	5.63*E* − 115
Collagen type I alpha 1 chain (*Col1a1*)	24.55	11.64	−1.08	1.25*E* − 51
Cytochrome P450, family 2, subfamily a, polypeptide 1 (*Cyp2a1*)	20.22	9.58	−1.08	2.40*E* − 12
DNA-damage-inducible transcript 4 (*Ddit4*)	19.42	9.41	−1.05	6.13*E* − 12
Collagen type III alpha 1 chain (*Col3a1*)	18.55	6.56	−1.50	2.34*E* − 53
Insulin-like growth factor binding protein 4 (*Igfbp4*)	12.38	5.52	−1.17	9.83*E* − 12
Coiled-coil domain containing 39 (*Ccdc39*)	6.10	3.02	−1.01	4.61*E* − 10
Core 1 synthase, glycoprotein-N-acetylgalactosamine 3-beta-galactosyltransferase, 1 (*C1galt1*)	6.69	3.01	−1.15	4.19*E* − 05
Secernin 1 (*Scrn1*)	12.61	2.69	−2.23	5.56*E* − 20

**Table 6:** Significantly upregulated and downregulated genes in both bone and adrenal gland (YD vs. blank).

Gene name	Bone			Adrenal gland				
	Expression level (FPKM)		log_2_ fold change (YD/blank)	*p* value	Expression level (FPKM)		log_2_ fold change (YD/blank)	*p* value
	Blank	YD			Blank	YD		
*Upregulation genes*								
Arachidonate 15-lipoxygenase (*Alox15*)	294.41	950.34	1.69	0	0.63	39.28	5.96	2.34*E* − 203
Family with sequence similarity 111, member A (*Fam111a*)	33.13	366.26	3.47	0	2.73	8.09	1.57	4.43*E* − 17
Radical S-adenosyl methionine domain containing 2 (*Rsad2*)	87.04	178.65	1.04	6.09*E* − 215	0.56	1.74	1.64	7.79*E* − 06
Asparagine synthetase (*Asns*)	69.83	145.14	1.06	7.38*E* − 121	3.16	7.58	1.26	4.32*E* − 11
Cysteine-rich with EGF-like domains 2 (*Creld2*)	26.37	73.91	1.49	1.58*E* − 51	6.74	20.49	1.60	7.17*E* − 18
DNA-damage-inducible transcript 3 (*Ddit3*)	8.19	18.49	1.17	1.37*E* − 06	7.83	18.76	1.26	4.51*E* − 08

*Downregulation genes*								
Hematopoietically expressed homeobox (*Hhex*)	14.77	5.44	−1.44	9.16*E* − 14	298.33	137.41	−1.12	1.02*E* − 178
Myosin X (*Myo10*)	4.42	2.15	−1.04	3.54*E* − 12	115.23	53.06	−1.12	≤0.001
ADAM metallopeptidase with thrombospondin type 1 motif, 9 (*Adamts9*)	4.20	1.54	−1.45	2.07*E* − 21	13.81	6.33	−1.13	7.29*E* − 47
Protein kinase C, epsilon (*Prkce*)	3.39	1.17	−1.53	1.87*E* − 07	5.02	2.41	−1.06	2.20*E* − 05
Thyroid hormone responsive (*Thrsp*)	6.34	0.61	−3.38	4.10*E* − 13	5.52	1.71	−1.69	2.83*E* − 06

## Data Availability

The data sets used and/or analyzed during the current study are available from the corresponding author on reasonable request.

## Abbreviations

TCM: Traditional Chinese medicine
YD: Kidney-yang deficiency
RNA-Seq: RNA sequencing
SPF: Specific pathogen-free
SD: Sprague Dawley
DEGs: Differentially expressed genes
ACTH: Adrenocorticotropic hormone
CORT: Cortisol
FSH: Follicle-stimulating hormone
T: Testosterone
T3: Triiodothyronine
T4: Thyroxine
KEGG: Kyoto Encyclopedia of Genes and Genomes
SRA: Sequence read archive
NCBI: National Center for Biotechnology Information
GO: Gene ontology
NR: Nonredundant.

## Supplementary Materials

Supplementary Materials
*Supplementary Table 1*. Significantly upregulated and downregulated genes in both cartilage and adrenal gland (YD vs. blank). *Supplementary Table 2*. Significantly upregulated and downregulated genes in both bone and thyroid (YD vs. blank). *Supplementary Table 3*. Significantly upregulated and downregulated genes in both cartilage and thyroid (YD vs. blank). *Supplementary Table 4*. List of genes and their specific primer sequences for qRT-PCR validation. *Supplementary Figures 1, 2, 3, and 4*. Histogram display of GO enrichment analysis of DEGs. 2e results are divided into three categories: cellular components, molecular functions, and biological processes. The 2e *x*-axis represents the number of DEGs corresponding to each GO term, and the *y*-axis represents the name of each GO term. *Supplementary Figures 5, 6, 7, and 8*. Scatter plot display of KEGG enrichment analysis of DEGs. The 2e *x*-axis represents each enrichment factor, representing the proportion of DEGs involved in each KEGG pathway among all identified DEGs, and the *y*-axis represents each enrichment pathway. The 2e size of the dot reflects the number of DEGs, and the color of the dot reflects the adjusted *p* value (Q value).
