# Supplementary material for: Integrated RNA-Seq Analysis Uncovers the Potential Mechanism of the “Kidney Governing Bones” Theory of TCM
Source: Evid Based Complement Alternat Med. 2022 Mar 30;2022:7044775. doi: 10.1155/2022/7044775 (PMC8986393; doi:10.1155/2022/7044775)
Supplement: Supplementary Materials — Supplementary Table 1. Significantly upregulated and downregulated genes in both cartilage and adrenal gland (YD vs. blank). Supplementary Table 2. Significantly upregulated and downregulated genes in both bone and thyroid (YD vs. blank). Supplementary Table 3. Significantly upregulated and downregulated genes in both cartilage and thyroid (YD vs. blank). Supplementary Table 4. List of genes and their specific primer sequences for qRT-PCR validation. Supplementary Figures 1, 2, 3, and 4. Histogram display of GO enrichment analysis of DEGs. 2e results are divided into three categories: cellular components, molecular functions, and biological processes. The 2e x-axis represents the number of DEGs corresponding to each GO term, and the y-axis represents the name of each GO term. Supplementary Figures 5, 6, 7, and 8. Scatter plot display of KEGG enrichment analysis of DEGs. The 2e x-axis represents each enrichment factor, representing the proportion of DEGs involved in each KEGG pathway among all identified DEGs, and the y-axis represents each enrichment pathway. The 2e size of the dot reflects the number of DEGs, and the color of the dot reflects the adjusted p value (Q value). [file 7044775.f1.docx]

Supplementary Fig. 1 GO enrichment analysis of differentially expressed genes in kidney under YD


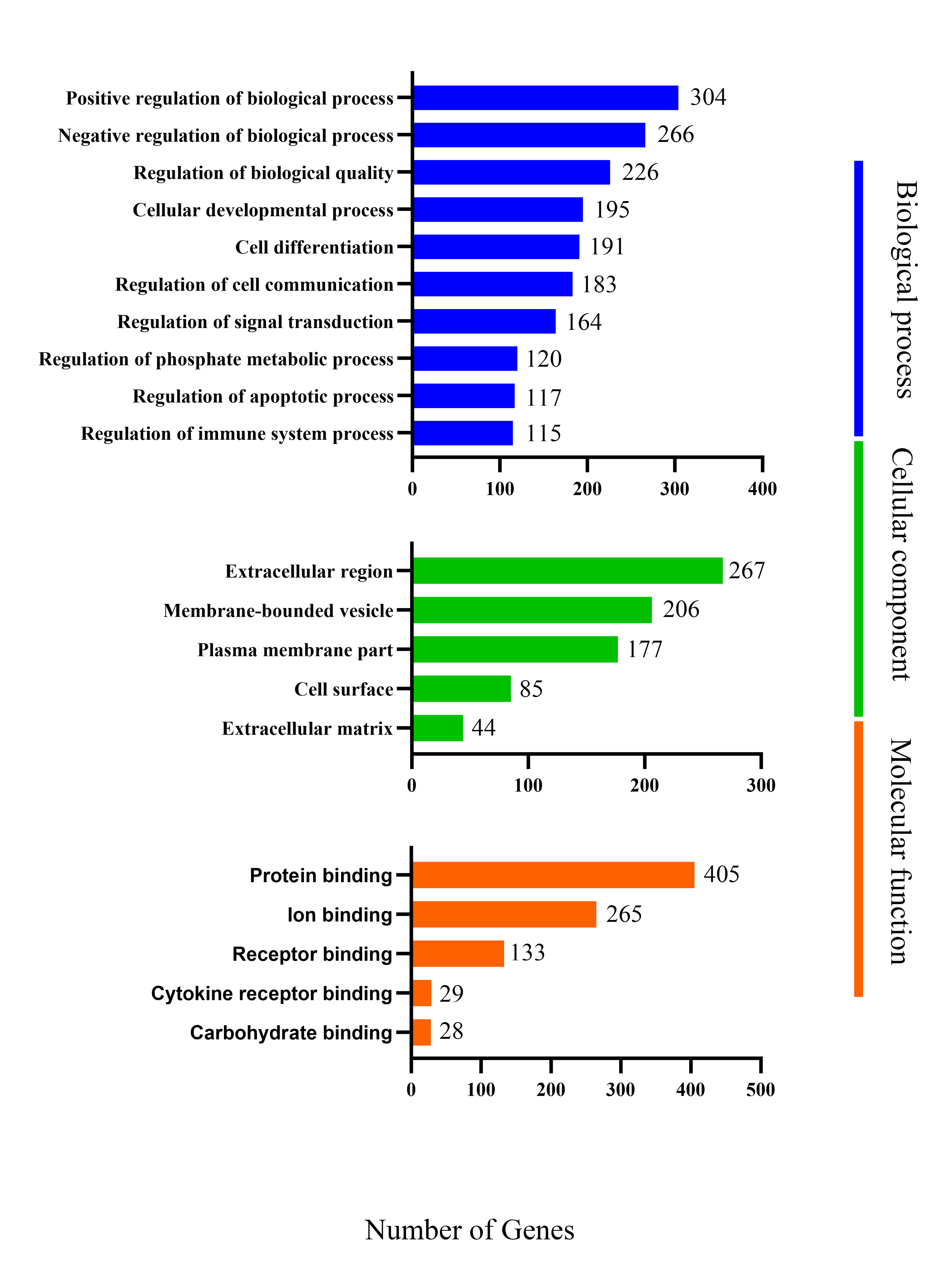


Histogram display of GO enrichment analysis of DEGs. 2e results are divided into three categories: cellular components, molecular functions, and biological processes. 2e x-axis represents the number of DEGs corresponding to each GO term, and the y-axis represents the name of each GO term.

Supplementary Fig. 2 GO enrichment analysis of differentially expressed genes in testis under YD


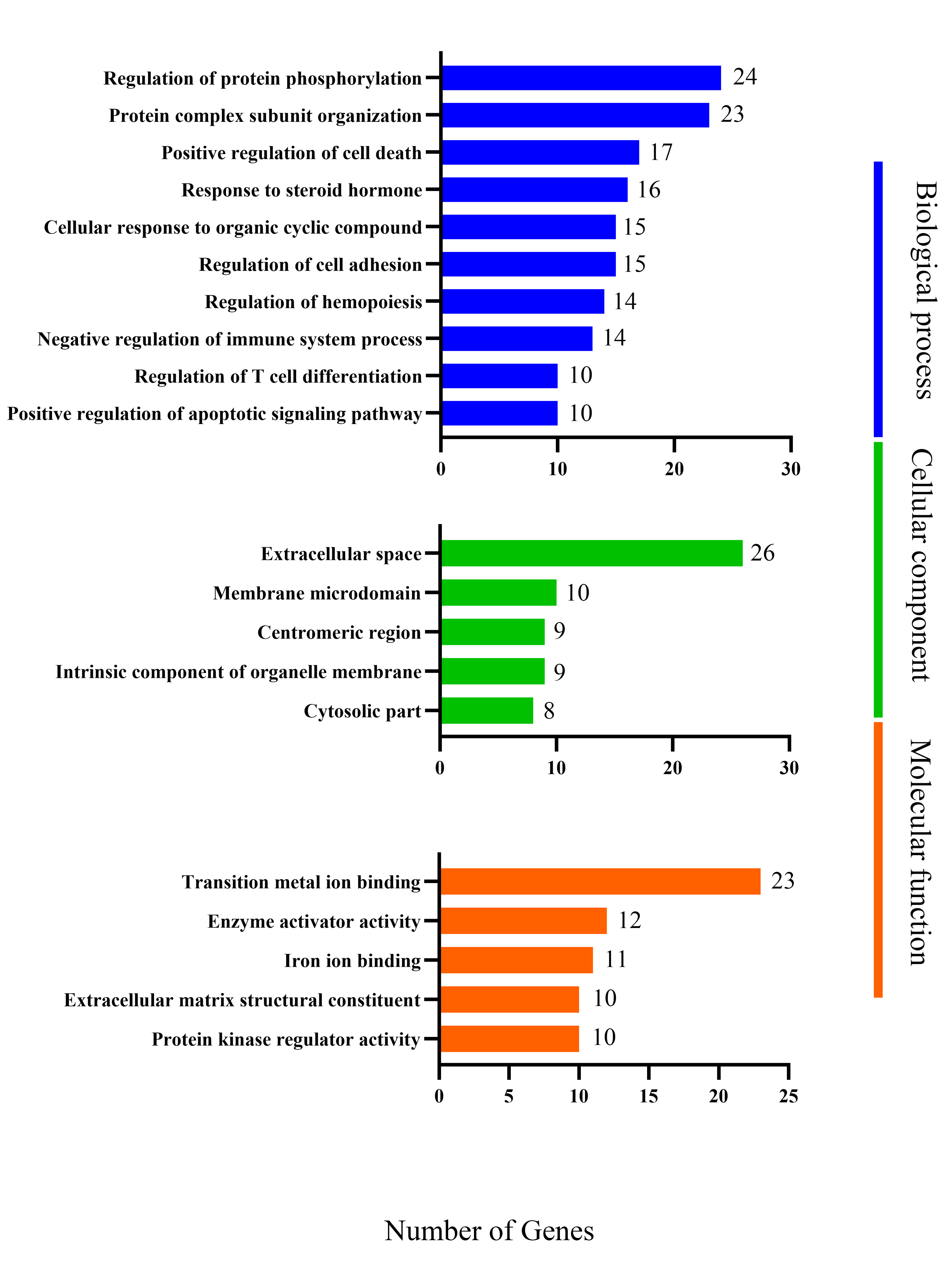


Histogram display of GO enrichment analysis of DEGs. 2e results are divided into three categories: cellular components, molecular functions, and biological processes. 2e x-axis represents the number of DEGs corresponding to each GO term, and the y-axis represents the name of each GO term.

Supplementary Fig. 3 GO enrichment analysis of differentially expressed genes in [adrenal](D:/LenovoSoftstore/Install/wangyiweidaocidian/8.9.6.0/resultui/html/index.html#/javascript:;) [gland](D:/LenovoSoftstore/Install/wangyiweidaocidian/8.9.6.0/resultui/html/index.html#/javascript:;) under YD


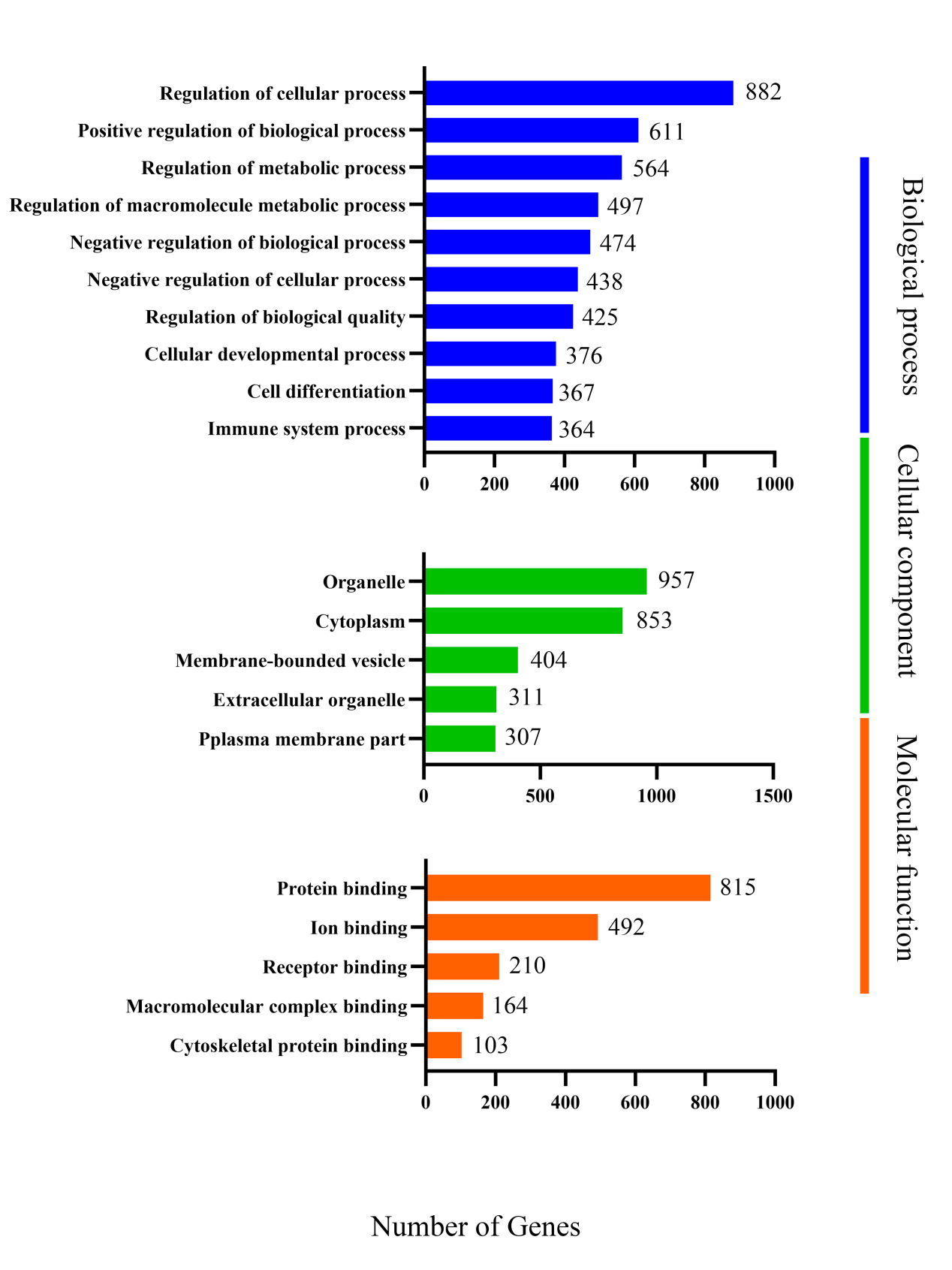


Histogram display of GO enrichment analysis of DEGs. 2e results are divided into three categories: cellular components, molecular functions, and biological processes. 2e x-axis represents the number of DEGs corresponding to each GO term, and the y-axis represents the name of each GO term.

Supplementary Fig. 4 GO enrichment analysis of differentially expressed genes in thyroid under YD


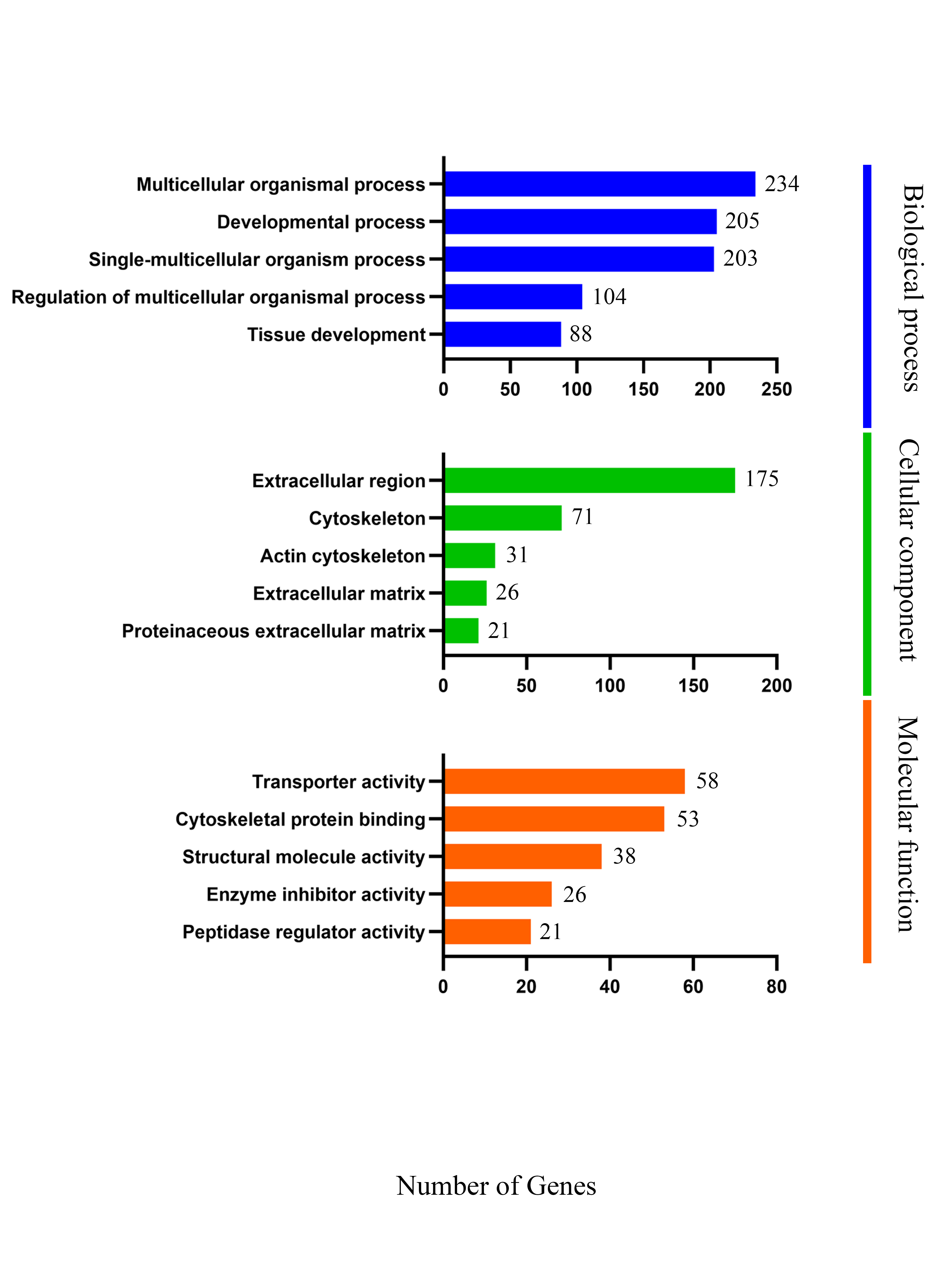


Histogram display of GO enrichment analysis of DEGs. 2e results are divided into three categories: cellular components, molecular functions, and biological processes. 2e x-axis represents the number of DEGs corresponding to each GO term, and the y-axis represents the name of each GO term.

Supplementary Fig. 5 KEGG enrichment analysis of differentially expressed genes in kidney under YD


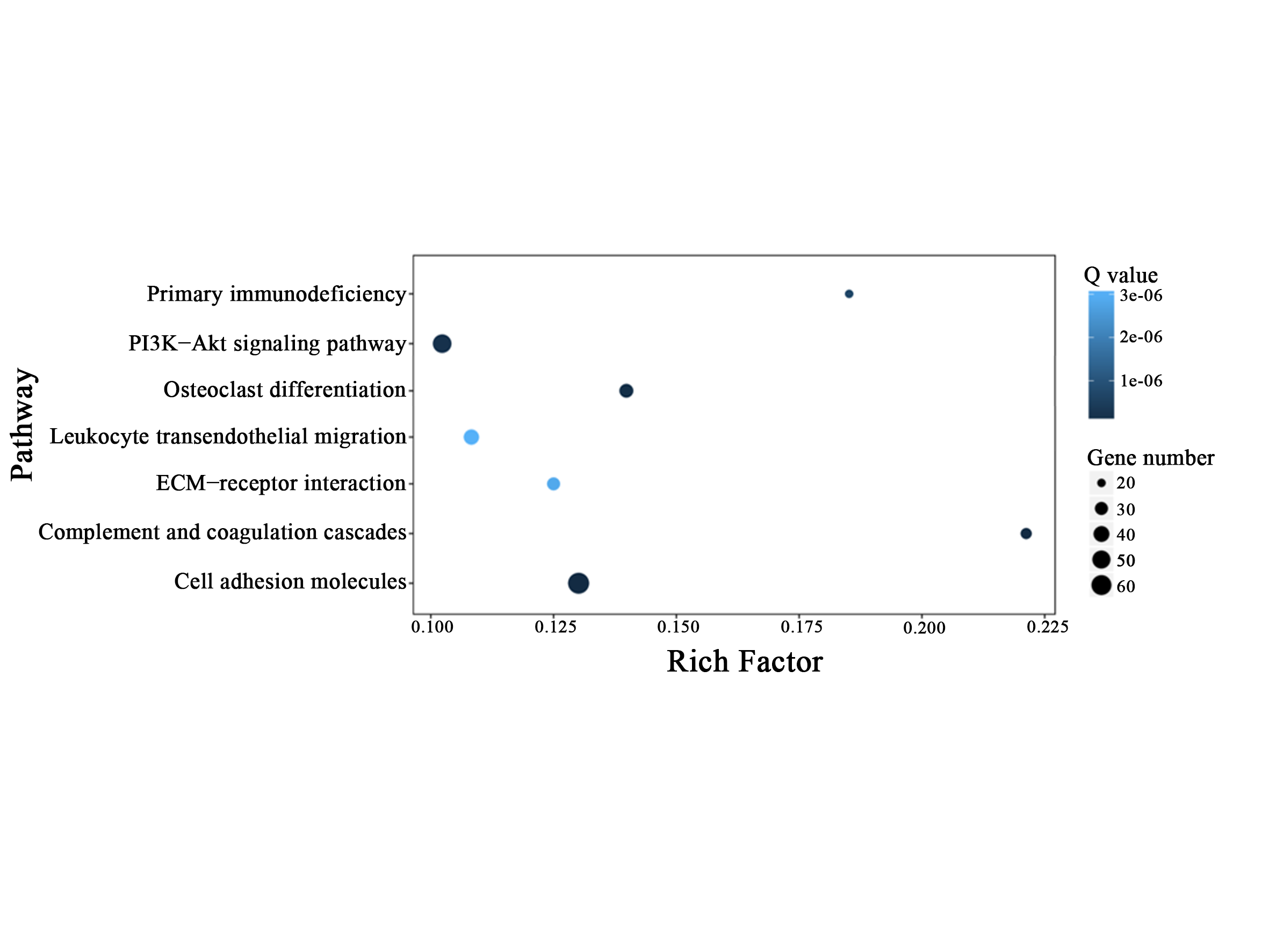


Scatter plot display of KEGG enrichment analysis of DEGs. 2e x-axis represents each enrichment factor, representing the proportion of DEGs involved in each KEGG pathway among all identified DEGs, and the y-axis represents each enrichment pathway. 2e size of the dot reflects the number of DEGs, and the color of the dot reflects the adjusted p value (Q value).

Supplementary Fig. 6 KEGG enrichment analysis of differentially expressed genes in testis under YD


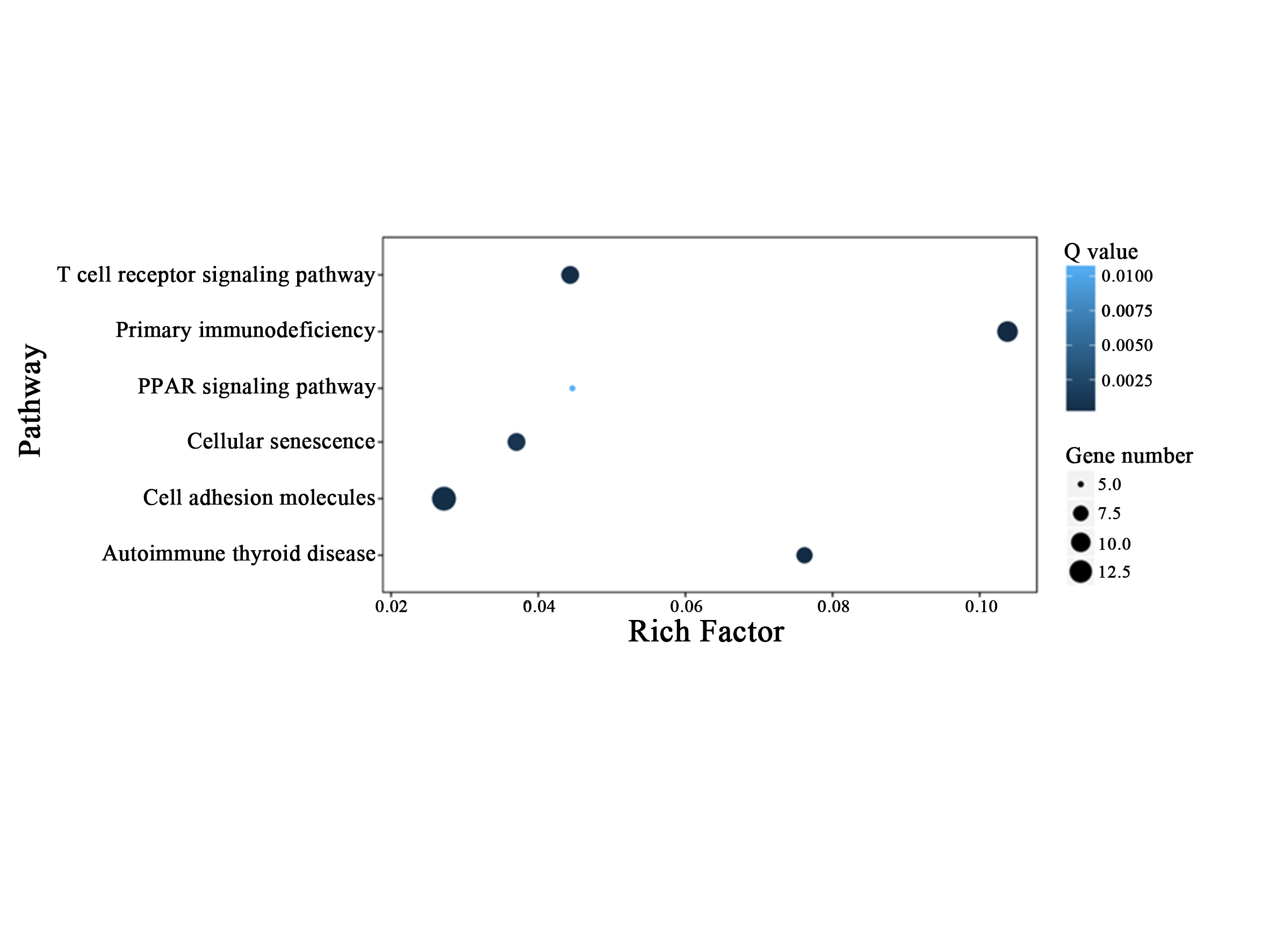


Scatter plot display of KEGG enrichment analysis of DEGs. 2e x-axis represents each enrichment factor, representing the proportion of DEGs involved in each KEGG pathway among all identified DEGs, and the y-axis represents each enrichment pathway. 2e size of the dot reflects the number of DEGs, and the color of the dot reflects the adjusted p value (Q value).

Supplementary Fig. 7 KEGG enrichment analysis of differentially expressed genes in [adrenal](D:/LenovoSoftstore/Install/wangyiweidaocidian/8.9.6.0/resultui/html/index.html#/javascript:;) [gland](D:/LenovoSoftstore/Install/wangyiweidaocidian/8.9.6.0/resultui/html/index.html#/javascript:;)  under YD


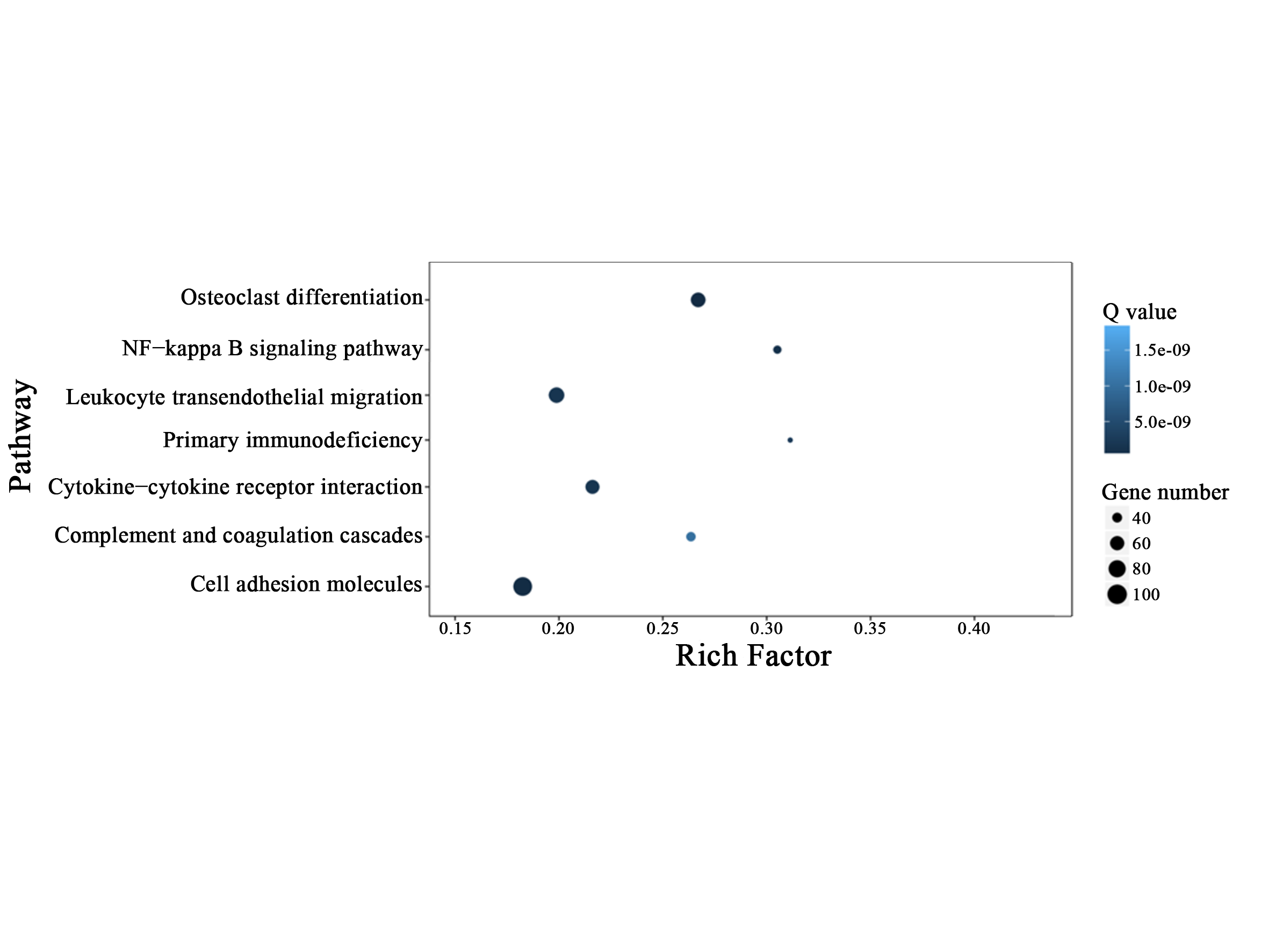


Scatter plot display of KEGG enrichment analysis of DEGs. 2e x-axis represents each enrichment factor, representing the proportion of DEGs involved in each KEGG pathway among all identified DEGs, and the y-axis represents each enrichment pathway. 2e size of the dot reflects the number of DEGs, and the color of the dot reflects the adjusted p value (Q value).

Supplementary Fig. 8 KEGG enrichment analysis of differentially expressed genes in thyroid under YD


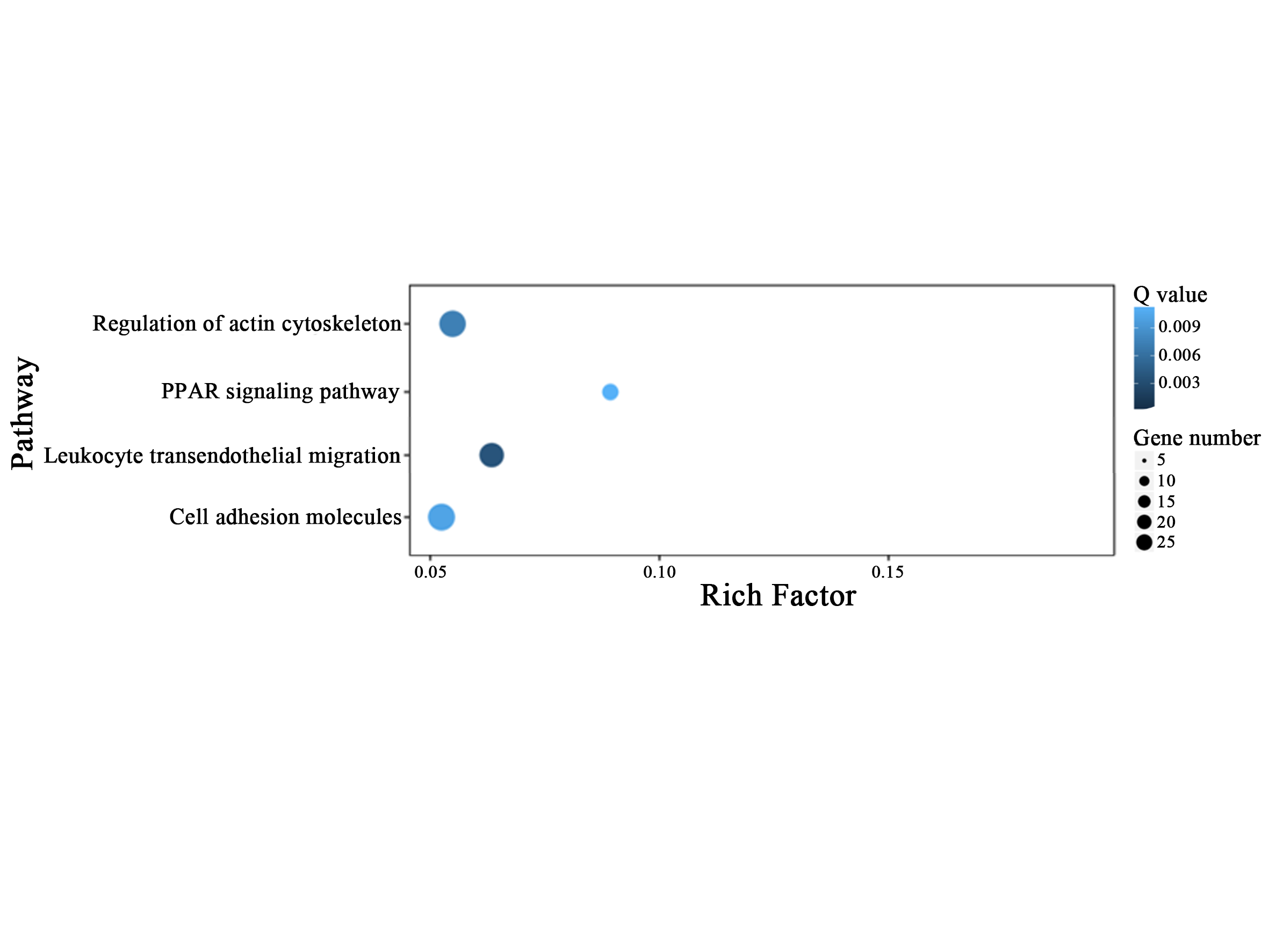


Scatter plot display of KEGG enrichment analysis of DEGs. 2e x-axis represents each enrichment factor, representing the proportion of DEGs involved in each KEGG pathway among all identified DEGs, and the y-axis represents each enrichment pathway. 2e size of the dot reflects the number of DEGs, and the color of the dot reflects the adjusted p value (Q value).

Supplementary Table 1 Significantly up-regulated and down-regulated genes in both cartilage and [adrenal](D:/LenovoSoftstore/Install/wangyiweidaocidian/8.9.6.0/resultui/html/index.html#/javascript:;) [gland](D:/LenovoSoftstore/Install/wangyiweidaocidian/8.9.6.0/resultui/html/index.html#/javascript:;) (YD vs. Blank)

| Gene name | Cartilage | | | | [Adrenal](D:/LenovoSoftstore/Install/wangyiweidaocidian/8.9.6.0/resultui/html/index.html#/javascript:;) [gland](D:/LenovoSoftstore/Install/wangyiweidaocidian/8.9.6.0/resultui/html/index.html#/javascript:;) | | | |
| --- | --- | --- | --- | --- | --- | --- | --- | --- |
|  | Expression level (FPKM) | | log_2_ fold change (YD/Blank) | p value | Expression level (FPKM) | | log_2_ fold change (YD/Blank) | p value |
|  | Blank | YD |  |  | Blank | YD |  |  |
| *Up-Regulation genes* | | | | | | | | |
| S100 calcium binding protein A9 (S100a9) | 569.32 | 14922.88 | 4.710 | 0 | 0.33 | 57.32 | 7.44 | 5.06E-51 |
| S100 calcium binding protein A8 (S100a8) | 448.93 | 12503.19 | 4.80 | 0 | 1.70 | 70.86 | 5.38 | 2.93E-38 |
| Carbonic anhydrase 2 (Car2) | 280.28 | 865.88 | 1.63 | 0 | 5.07 | 12.84 | 1.34 | 2.73E-08 |
| Cytochrome b-245 alpha chain (Cyba) | 191.89 | 467.10 | 1.28 | 2.05E-109 | 19.11 | 86.96 | 2.19 | 4.06E-53 |
| Arachidonate 15-lipoxygenase（Alox15） | 52.07 | 234.36 | 2.17 | 0 | 0.63 | 39.28 | 5.96 | 2.34E-203 |
| Matrix metallopeptidase 8 (Mmp8) | 25.54 | 149.76 | 2.55 | 8.03E-214 | 0.01 | 1.39 | 7.12 | 1.57E-05 |
| *Down-Regulation genes* | | | | | | | | |
| Insulin induced gene 1 (Insig1) | 48.27 | 23.89 | -1.01 | 8.36E-36 | 234.36 | 83.25 | -1.49 | ≤ 0.001 |
| Nuclear receptor subfamily 1, group D, member 1 (Nr1d1) | 49.35 | 13.53 | -1.87 | 2.40E-86 | 57.40 | 27.89 | -1.04 | 8.62E-48 |
| Solute carrier family 26 member 2 (Slc26a2) | 23.86 | 8.90 | -1.42 | 3.85E-103 | 7.26 | 3.21 | -1.18 | 8.54E-28 |
| Insulin-like growth factor 1 (Igf1) | 25.01 | 8.24 | -1.60 | 5.81E-28 | 33.55 | 12.97 | -1.37 | 1.97E-32 |
| Peroxisome proliferator-activated receptor delta (Ppard) | 17.27 | 7.26 | -1.25 | 2.37E-25 | 100.42 | 45.77 | -1.13 | 3.23E-145 |
| ADAM metallopeptidase with thrombospondin type 1 motif, 9 (Adamts9) | 12.20 | 3.71 | -1.72 | 2.25E-71 | 13.81 | 6.33 | -1.13 | 7.29E-47 |
| Plasminogen activator, tissue type (Plat) | 8.25 | 1.93 | -2.10 | 6.33E-20 | 67.86 | 33.90 | -1.00 | 2.11E-58 |
| Laminin subunit alpha 5 (Lama5) | 1.06 | 0.48 | -1.14 | 6.19E-06 | 9.18 | 3.27 | -1.49 | 8.40E-68 |

Supplementary Table 2 Significantly up-regulated and down-regulated genes in both bone and thyroid (YD vs. Blank)

| Gene name | Cartilage | | | | [Adrenal](D:/LenovoSoftstore/Install/wangyiweidaocidian/8.9.6.0/resultui/html/index.html#/javascript:;) [gland](D:/LenovoSoftstore/Install/wangyiweidaocidian/8.9.6.0/resultui/html/index.html#/javascript:;) | | | |
| --- | --- | --- | --- | --- | --- | --- | --- | --- |
|  | Expression level (FPKM) | | log_2_ fold change (YD/Blank) | p value | Expression level (FPKM) | | log_2_ fold change (YD/Blank) | p value |
|  | Blank | YD |  |  | Blank | YD |  |  |
| *Up-Regulation genes* | | | | | | | | |
| Arachidonate 15-lipoxygenase (Alox15) | 294.41 | 950.34 | 1.69 | 0 | 1.40 | 62.44 | 5.48 | 5.86E-306 |
| Radical S-adenosyl methionine domain containing 2 (Rsad2) | 87.04 | 178.65 | 1.04 | 6.09E-215 | 0.73 | 2.41 | 1.72 | 5.60E-08 |
| B-cell CLL/lymphoma 3(Bcl3) | 9.30 | 19.10 | 1.04 | 3.09E-12 | 1.64 | 6.44 | 1.97 | 2.45E-11 |
| Gremlin 1, DAN family BMP antagonist (Grem1) | 1.28 | 4.06 | 1.67 | 4.16E-12 | 0.18 | 1.37 | 2.93 | 8.14E-09 |
| Cytokine like 1 (Cytl1) | 0.40 | 2.62 | 2.71 | 3.27E-07 | 9.51 | 35.45 | 1.90 | 9.82E-56 |
| Hyaluronan and proteoglycan link protein 1 (Hapln1) | 0.06 | 1.53 | 4.67 | 1.16E-07 | 0.01 | 1.93 | 7.59 | 1.97E-11 |
| *Down-Regulation genes* | | | | | | | | |
| Collagen type I alpha 1 chain (Col1a1) | 501.94 | 132.60 | -1.92 | 0 | 60.63 | 20.07 | -1.59 | 5.13E-244 |
| Collagen type I alpha 2 chain (Col1a2) | 388.58 | 84.83 | -2.20 | 0 | 58.20 | 27.77 | -1.07 | 1.30E-96 |
| Collagen type III alpha 1 chain (Col3a1) | 29.78 | 12.99 | -1.20 | 8.51E-62 | 140.76 | 46.36 | -1.60 | ≤ 0.001 |

Supplementary Table 3 Significantly up-regulated and down-regulated genes in both cartilage and thyroid (YD vs. Blank)

| Gene name | Cartilage | | | | [Adrenal](D:/LenovoSoftstore/Install/wangyiweidaocidian/8.9.6.0/resultui/html/index.html#/javascript:;) [gland](D:/LenovoSoftstore/Install/wangyiweidaocidian/8.9.6.0/resultui/html/index.html#/javascript:;) | | | |
| --- | --- | --- | --- | --- | --- | --- | --- | --- |
|  | Expression level (FPKM) | | log_2_ fold change (YD/Blank) | p value | Expression level (FPKM) | | log_2_ fold change (YD/Blank) | p value |
|  | Blank | YD |  |  | Blank | YD |  |  |
| *Up-Regulation genes* | | | | | | | | |
| S100 calcium binding protein A9 (S100a9) | 569.32 | 14922.88 | 4.71 | 0 | 4.89 | 26.42 | 2.43 | 1.04E-11 |
| S100 calcium binding protein A8 (S100a8) | 448.93 | 12503.19 | 4.80 | 0 | 5.42 | 35.68 | 2.72 | 1.57E-11 |
| Cytokine like 1 (Cytl1) | 340.39 | 1736.51 | 2.35 | 0 | 9.51 | 35.45 | 1.90 | 9.82E-56 |
| Secretory leukocyte peptidase inhibitor (Slpi） | 18.35 | 69.12 | 1.91 | 3.30E-28 | 6.12 | 170.92 | 4.80 | 2.70E-187 |
| Phospholipase A2 group IIA (Pla2g2a) | 4.20 | 55.62 | 3.73 | 2.25E-57 | 2.55 | 11.44 | 2.17 | 7.92E-09 |
| *Down-Regulation genes* | | | | | | | | |
| Collagen type I alpha 1 chain (Col1a1) | 3997.45 | 253.96 | -3.98 | 0 | 60.63 | 20.07 | -1.59 | 5.13E-244 |
| Collagen type I alpha 2 chain (Col1a2) | 2560.38 | 178.99 | -3.84 | 0 | 58.20 | 27.77 | -1.07 | 1.30E-96 |
| Collagen type III alpha 1 chain (Col3a1) | 89.46 | 9.08 | -3.30 | 0 | 140.76 | 46.36 | -1.60 | ≤ 0.001 |
| Collagen type XV alpha 1 chain (Col15a1) | 18.32 | 1.68 | -3.45 | 1.22E-147 | 29.41 | 14.26 | -1.04 | 8.97E-56 |
| Secreted frizzled-related protein 2 (Sfrp2) | 9.53 | 0.07 | -7.09 | 7.10E-42 | 4.45 | 1.78 | -1.32 | 8.96E-06 |

Supplementary Table 4 List of genes and their specific primer sequences for qRT-PCR validation

| Gene name | Primer | Sequence |
| --- | --- | --- |
| Col1a1 | Forward primer | AGCCGCAAAGAGTCTACATG |
|  | Reverse primer | CTTAGGCCATTGTGTATGCAG |
| Ibsp | Forward primer | CTGACGCTGGAAAGTTGGAG |
|  | Reverse primer | CGTTGACGACCTGCTCATTT |
| Bglap | Forward primer | GACCCTCTCTCTGCTCACTC |
|  | Reverse primer | GGGCTCCAAGTCCATTGTTG |
| Dcn | Forward primer | GAGCCTTGCAGGGAATGAAG |
|  | Reverse primer | GTTGCCATCCAGATGCAGTT |
| Dmp1 | Forward primer | ATGACAGCCAGGATGTGGAA |
|  | Reverse primer | TTCCTCCTTGGAGCGGAAAT |
| Sost | Forward primer | TCAGAGAGTACCCAGAGCCT |
|  | Reverse primer | TACTCGGACACGTCTTTGGT |
| Phex | Forward primer | CCTCCTACCAGGCATCACAT |
|  | Reverse primer | CAGCTTCCGGTCTGTAGGAA |
| Mepe | Forward primer | AGGAGAACAAGCCACCCTAC |
|  | Reverse primer | ACTGCTCTCGCTAGAACTCC |
| Col2a1 | Forward primer | CAAGAAGGCCTTGCTCATCC |
|  | Reverse primer | CAGTGTACGTGAACCTGCTG |
| Acan | Forward primer | TGCTTCTCCAGAAGGGTCAG |
|  | Reverse primer | TCAGACAAGGGCTTGAGAGG |
| Hapln1 | Forward primer | AGTGTTTCTGAAGGGAGGCA |
|  | Reverse primer | AGGGAACACCACACCTTGTA |
| Wwp2 | Forward primer | GATCACAACACCCGCACTAC |
|  | Reverse primer | TAGGGCATTTGAGTGGCAGA |
| Comp | Forward primer | CAGCTCAAGGCTGTCAAGTC |
|  | Reverse primer | CTTCCAGCCCACATTTCGAG |
| Fgfr3 | Forward primer | TGCTGGTGACTGAGGACAAT |
|  | Reverse primer | GAGGACACCAAAGGACCAGA |
| Pth1r | Forward primer | TCAGGGACATTGTGGCAGAT |
|  | Reverse primer | TAGCTGCTACTCCCACTTCG |
| Gapdh | Forward primer | CCATCAACGACCCCTTCATT |
|  | Reverse primer | GACCAGCTTCCCATTCTCAG |
